# Different types of cultured human adult Cardiac Progenitor Cells have a high degree of transcriptome similarity

**DOI:** 10.1111/jcmm.12458

**Published:** 2014-10-14

**Authors:** Roberto Gaetani, Dries A M Feyen, Pieter A Doevendans, Hendrik Gremmels, Elvira Forte, Joost O Fledderus, Faiz Z Ramjankhan, Elisa Messina, Mark A Sussman, Alessandro Giacomello, Joost P G Sluijter

**Affiliations:** a Dept. of Cardiology, DH&L, University Medical Center UtrechtUtrecht, The Netherlands; b Dept. of Molecular Medicine, Cenci-Bolognetti Foundation-Pasteur Institute, “Sapienza” University of RomeRome, Italy; c Interuniversity Cardiology Institute of the Netherlands (ICIN)Utrecht, The Netherlands; d Dept. of Nephrology and Hypertension, University Medical Center UtrechtUtrecht, The Netherlands; e Dept. of Cardio-Thoracic Surgery, DH&L, University Medical Center UtrechtUtrecht, The Netherlands; f Heart Institute, San Diego State UniversitySan Diego, CA, USA

The discovery and isolation of different resident cardiac progenitor cells (CPCs) a decade ago, as described by several research groups, stimulated the use of these cells for cardiac regeneration. Human CPCs are moving towards the clinic as one of the most promising cell types for cardiac repair, but the extent to which their molecular profiles vary as a result of donor heterogeneity or different isolation methods remain unclear. Defining a common molecular profile that defines CPC’s is therefore an important goal. Similarly, identifying robust and multilaboratory isolation and culture protocols that generate reproducible cell populations from genetically diverse donors is critical for their translational success.

In this respect, we collected human auricle biopsy samples anonymously from 20 different adult patients that underwent bypass surgery and generated a total number of 33 different cardiac derived progenitor cell (CPC) lines (Table S1). Human CPCs were isolated according the original published protocol, based on c-kit 1 or Sca-1 2 expression or auricles were cut in 1 mm^3^ parts and cultured as explants to obtain Cardiospheres (CSps) 3 and Cardiosphere Derived Cells (CDCs) 4. CPCs were subsequently propagated in a panel of different media formulations, either in their originally described culture media or switched to media and culture coatings of the other CPC subsets (Figure S1, Table S7). When comparing individual CPC cell-lines, isolated with different methodologies, they shared a high degree of similarities and correlation in gene expression patterns (Fig. 1B). By averaging expression profiles of individual CPC conditions, thereby reducing donor variability, similarities increased even more, ranging from 0.92 to 0.96 (Fig. 1C; Table S2). These results suggest that individual donor differences were larger than influences of isolation and medium conditions. Moreover, the strongest correlations between the different CPC lines were observed when cells were isolated and cultured in the same conditions. Among the different CPCs, spheres-growing CSps showed the least correlation (0.91–0.96), while monolayer-growing CPCs shared higher correlations among them (0.96–0.98). We performed a moderated *t*-test to evaluate significant differentially expressed genes between the individual samples (Tables S3 and S4). Out of the 13,073 analysd genes, we found only few genes differentially expressed in 5 of 20 different monolayer-cultured CPC cell-lines comparisons. Only when the 3D-cultured CSps were compared with the other CPCs more differently expressed genes could be identified. Although only limited genes were different, we further explored if we could identify differences in gene patterns between the different CPC populations, based on selected genes important for stem cell-maintenance, their growth and biology. In particular, we evaluated genes involved in the regulation of different stem cell pathways like TGF-β, Wnt, NFkB, p53, JAK/STAT, Notch and Hedgehog (Fig. S2A), cell cycle (Fig. S2B), stem cell transcription factors (Fig. S2C), and growth factors, cytokines and chemokines (Fig. S2D). Detailed heat map analysis showed again; however, a very similar profile among all samples, with small differences mainly related to individual donors and not to different cell types or conditions (Fig. S2). Since CSps and monolayer growing CPCs have differently expressed patterns, we selected all the significantly differentially expressed genes that displayed a two fold or more difference and compared them with CDCs, and c-Kit and Sca-1+ CPCs monolayer-cultures (Table S6). Ingenuity pathway analysis identified a gene network in CSps that is enriched in genes encoding for growth factor production and signalling molecules involved in the development of cardiac muscle, vasculogenesis and angiogenesis (Fig. 2). Among them BMP-2, HGF, LIF, PTGS-2, VEGFA and PDGFRB are known to play an important role during cardiac development. Moreover, having a protective effect on a developing heart failure.

**Figure 1:**
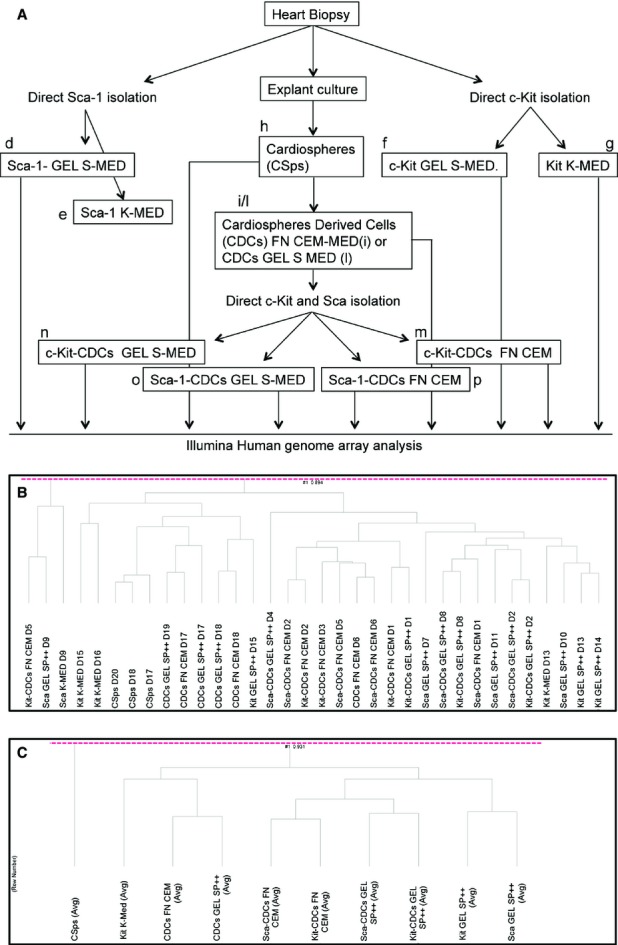
Experimental design of the project (**A**) and hierarchical clustering of CPCs samples (**B** and **C**). Sca-1+ cells were isolated from human auricle biopsy and cultured in gelatin coated flask and Sca-1 medium (Sca GEL S-MED) (2) (d). After expansion cells were also cultured in c-Kit culture condition (Sca K-MED) (1) (e). C-Kit+ cells were isolated and cultured in Kit-CPCs Medium (Kit K-MED) (1) (f) and after expansion cultured in gelatin coated flask and Sca-1 medium (Kit- GEL S-MED) (2) (g). Human auricle samples were cultured as explant to form Cardiospheres (CSps) (3) (h). CSPs were expanded as Cardiospheres derived cells (CDCs) in Fibronectin and Complete Explant Medium (FN CEM) (4) (i) or in gelatin coated flasks and Sca medium (CDCs GEL S-MED) (2) (l). After expansion c-kit+ (m/n) or Sca-1+ (o/p) were isolated from CDCs FN CEM and cultured or in FN CEM (Kit-CDCs FN CEM (m) or Sca-CDCs FN CEM (p)) or in GEL S-MED condition (Kit-CDCs GEL S-MED (n) or Sca-CDCs GEL S-MED (o). (**B**) Hierarchical clustering of all different CPCs samples. (**C**) Hierarchical clustering of CPCs samples, averaged per isolation and culture condition.

**Figure 2:**
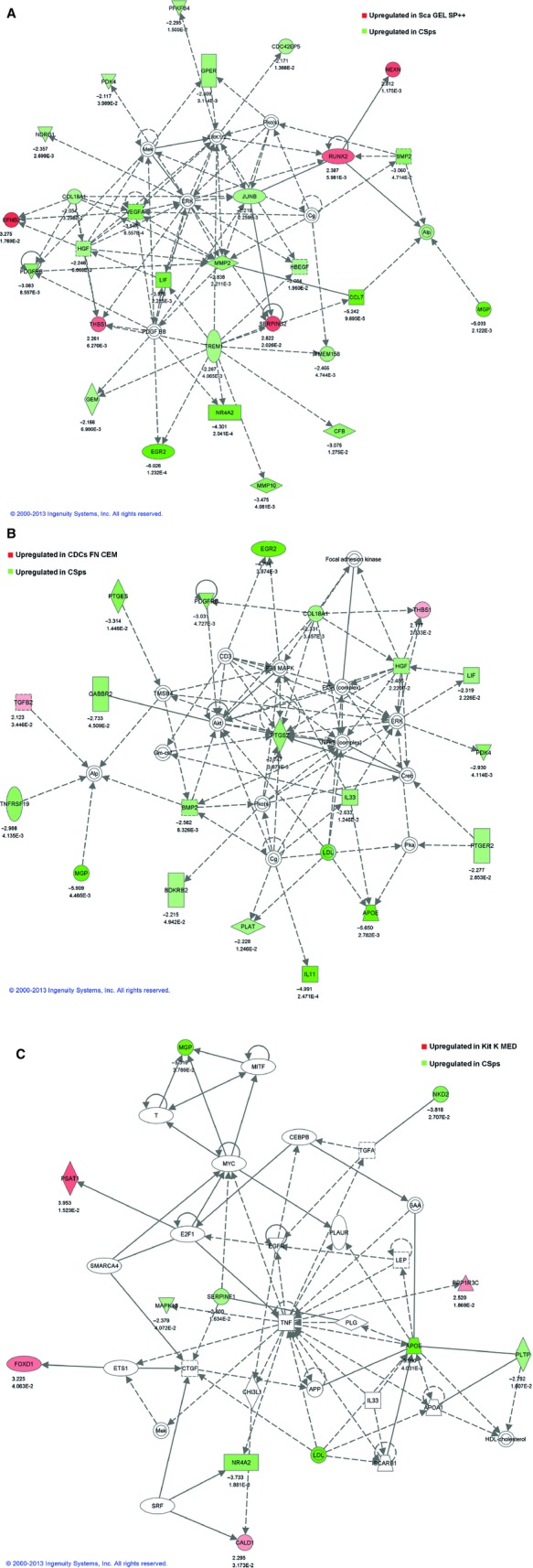
Ingenuity molecular networks analysis of the differentially expressed genes. Fold difference ≥2; p<0.05. (**A**) Differentially regulated genes between CSps and Sca GEL SP++ in Cardiovascular System Development and Function, Embryonic Development, Organismal Development network. (**B**) Differentially regulated genes between CSps *versus* CDCs FN CEM in Cardiovascular System Development and Function, Organismal Development, Cell-To-Cell Signaling and Interaction network. (**C**) Differentially regulated genes between CSps and Kit K-Med in Cardiovascular System Development and Function, Organismal Development, Tissue Morphology network.

Taken together, our data suggest that human CPCs can be isolated from patient heart biopsies using different markers, such as c-kit or Sca-1- like, and alternative methodologies, *via* direct cell isolation or *via* explant culture, such as CSps and CDCs. For the first time, however, we showed that upon culture expansion, these cell populations have a very similar gene expression profile, even more pronounced when cultured in comparable culture conditions and even transcended by donor differences. Among the different CPCs analysed, CSps are the most different, probably because of the unselected cell populations and containing more supporting cell population that form CSps and their particular 3D culture structure and thereby different interactions and growing conditions. Surprisingly CDCs, which is a cell population derived from CSps, are more similar with other antigen selected CPCs rather than with CSps, confirming the idea that monolayer and high proliferative culture condition might play an important role in minimizing the differences among the different CPCs analysed. Recently, Dey *et al*. isolated murine CPCs, based on different surface markers 5, and showed that these, non-cultured cells, represent progenitor cell populations at different stages of cardiac commitment 5. In our study, we did not observe such differences between the different human monolayer CPCs population upon culture propagation. A similar stage difference, however, might be present *in situ* in humans as well but lost upon culture expansion. The expression of these different stem cell markers and their co-expression probably represent different developmental and/or physiological stages of CPCs, rather than intrinsic different CPC populations. For future translation for cardiac cell therapy, our results suggest that we need to take into account the cell donor variability between patients more than the isolation methodology, and further study the correlation between CPC characteristics and *e.g*. the diseased status of a patient. Our findings are of fundamental importance to create a consensus among different scientists in the field of myocardial regeneration, which should help align future clinical approaches to improve the reported beneficial effects of cell therapy for heart disease by using cardiac derived progenitor cell populations.

## Supporting Information

Figure S1 Representative pictures of individual cultures are presented for each used condition.

Figure S2 Genome guided analysis of CPCs samples.

Table S1 CPCs samples and donors used in the project.

Table S2 pair-waise comparison of CPCs averaged values.

Table S3 (a) Significantly Differentially expressed genes between monolayer growing CPCs groups. (b) Significantly differentially expressed genes between CSps and monolayer growing CPCs.

Table S4 List of significantly differentially expressed genes between monolayer growing CPCs.

Table S5 (a) List of significantly differentially expressed genes between CSps and Sca GEL SP++. (b) List of significantly differentially expressed genes between CSps and CDCs FN CEM. (c) List of significantly differentially expressed genes between CSps and Kit K-MED.

Table S6 Ingenuity molecular networks analysis gene list.

Table S7 Culture conditions of Isolated CPCs.
