# Supplementary figures and images for: Different types of cultured human adult Cardiac Progenitor Cells have a high degree of transcriptome similarity
Source: J Cell Mol Med. 2014 Oct 14;18(11):2147–51. doi: 10.1111/jcmm.12458 (PMC4224548; doi:10.1111/jcmm.12458)

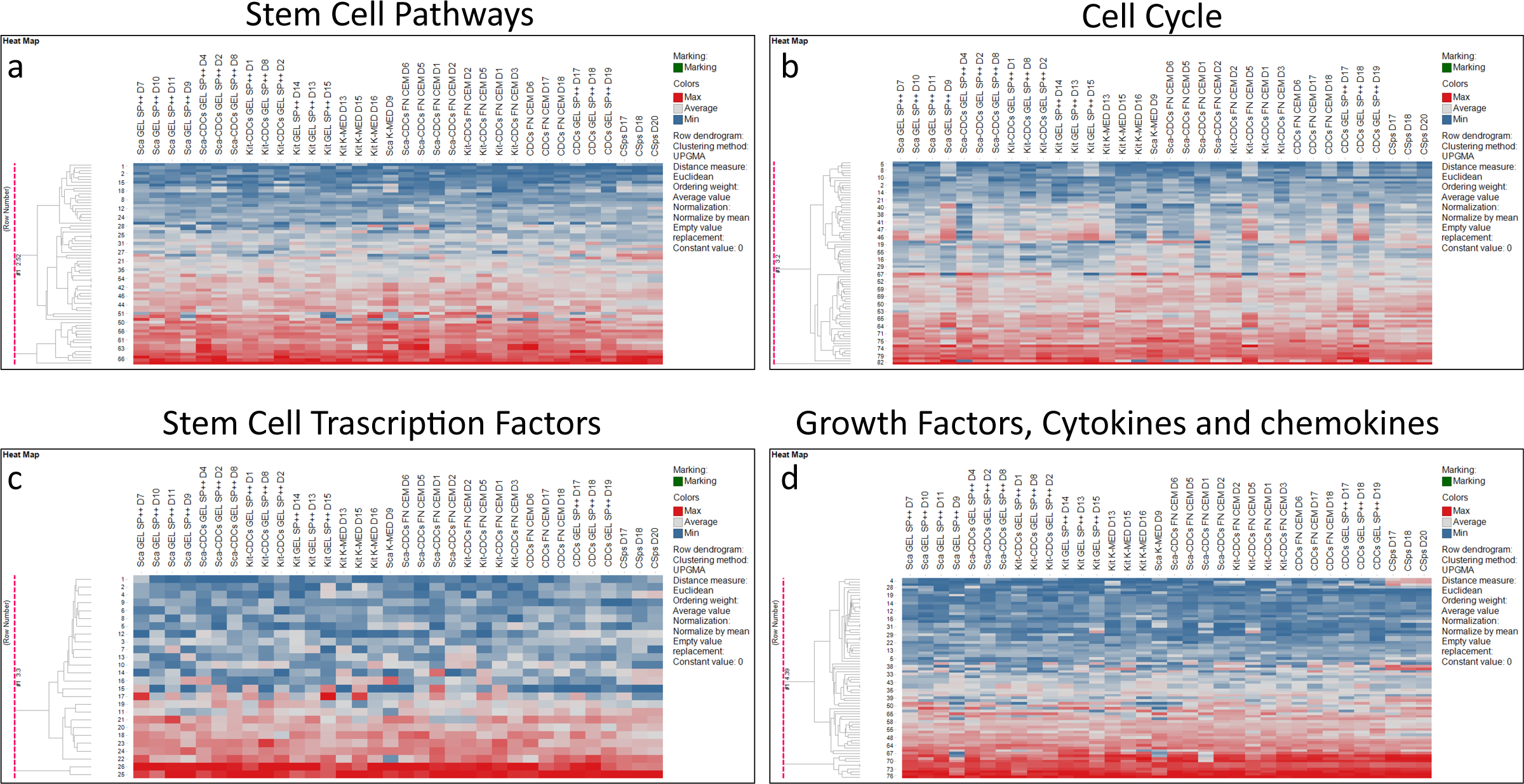

Supplement: Figure S2 — Genome guided analysis of CPCs samples. [file jcmm0018-2147-sd2.tif]
