# Supplementary material for: Different types of cultured human adult Cardiac Progenitor Cells have a high degree of transcriptome similarity
Source: J Cell Mol Med. 2014 Oct 14;18(11):2147–51. doi: 10.1111/jcmm.12458 (PMC4224548; doi:10.1111/jcmm.12458)
Supplement: Table S1 — CPCs samples and donors used in the project. [file jcmm0018-2147-sd3.doc]

**Supplementary Table 1:** CPCs samples and donors used in the project.

| **Cell**  **type**  **Donor**  **number** | **Kit-CDCs GEL SP++** | **Kit-CDCs FN CEM** | **Sca-CDCs GEL SP++** | **Sca-CDCs FN CEM** | **Sca GEL SP++** | **Kit GEL SP++** | **Kit K-Med** | **CDCs FN CEM** | **CDCs**  **GEL SP++** | **CSps** |
| --- | --- | --- | --- | --- | --- | --- | --- | --- | --- | --- |
| **D1** | X | X |  | X |  |  |  |  |  |  |
| **D2** | X | X | X | X |  |  |  |  |  |  |
| **D3** |  | X |  |  |  |  |  |  |  |  |
| **D4** |  |  | X |  |  |  |  |  |  |  |
| **D5** |  | X |  | X |  |  |  |  |  |  |
| **D6** |  |  |  | X |  |  |  | X |  |  |
| **D7** |  |  |  |  | X |  |  |  |  |  |
| **D8** | X |  | X |  |  |  |  |  |  |  |
| **D9** |  |  |  |  | X |  |  |  |  |  |
| **D10** |  |  |  |  | X |  |  |  |  |  |
| **D11** |  |  |  |  | X |  |  |  |  |  |
| **D12** |  |  |  |  |  |  |  |  |  |  |
| **D13** |  |  |  |  |  | X | X |  |  |  |
| **D14** |  |  |  |  |  | X |  |  |  |  |
| **D15** |  |  |  |  |  | X | X |  |  |  |
| **D16** |  |  |  |  |  |  | X |  |  |  |
| **D17** |  |  |  |  |  |  |  | X | X | X |
| **D18** |  |  |  |  |  |  |  | X | X | X |
| **D19** |  |  |  |  |  |  |  |  | X |  |
| **D20** |  |  |  |  |  |  |  |  |  | X |
| **Total analyzed samples 33** | **3** | **4** | **3** | **4** | **4** | **3** | **3** | **3** | **3** | **3** |
