# Supplementary material for: Different types of cultured human adult Cardiac Progenitor Cells have a high degree of transcriptome similarity
Source: J Cell Mol Med. 2014 Oct 14;18(11):2147–51. doi: 10.1111/jcmm.12458 (PMC4224548; doi:10.1111/jcmm.12458)
Supplement: Table S2 — pair-waise comparison of CPCs averaged values. [file jcmm0018-2147-sd4.doc]

**Supplementary Table 2**: pair-waise comparison of CPCs averaged values.

| **CPCs sample** | **Sca GEL SP++** | **Sca-CDCs GEL/SP++** | **Kit-CDCs GEL/SP++** | **Kit**  **GEL/SP++** | **Kit**  **K-Med** | **Sca-CDCs FN/CEM** | **Kit-CDCs FN/CEM** | **CDCs FN CEM** | **CDCs**  **GEL SP++** | **CSps** |
| --- | --- | --- | --- | --- | --- | --- | --- | --- | --- | --- |
| **Sca GEL SP++** | - | 0.976 | 0.974 | 0.986 | 0.951 | 0.966 | 0.98 | 0.956 | 0.953 | 0.912 |
| **Sca-CDCs GEL/SP++** | 0.976 | - | 0.987 | 0.968 | 0.959 | 0.98 | 0.98 | 0.962 | 0.96 | 0.923 |
| **Kit-CDCs GEL/SP++** | 0.974 | 0.987 | - | 0.967 | 0.954 | 0.981 | 0.984 | 0.966 | 0.958 | 0.92 |
| **Kit**  **GEL/SP++** | 0.986 | 0.968 | 0.967 | - | 0.954 | 0.958 | 0.972 | 0.961 | 0.964 | 0.917 |
| **Kit**  **K-Med** | 0.951 | 0.959 | 0.954 | 0.954 | - | 0.966 | 0.961 | 0.971 | 0.969 | 0.946 |
| **Sca-CDCs FN/CEM** | 0.966 | 0.98 | 0.981 | 0.958 | 0.966 | - | 0.988 | 0.978 | 0.962 | 0.925 |
| **Kit-CDCs FN/CEM** | 0.98 | 0.98 | 0.984 | 0.972 | 0.961 | 0.988 | - | 0.974 | 0.959 | 0.923 |
| **CDCs FN CEM** | 0.956 | 0.962 | 0.966 | 0.961 | 0.971 | 0.978 | 0.974 | - | 0.987 | 0.953 |
| **CDCs**  **GEL SP++** | 0.953 | 0.96 | 0.958 | 0.964 | 0.969 | 0.962 | 0.959 | 0.987 | - | 0.961 |
| **CSps** | 0.912 | 0.923 | 0.92 | 0.917 | 0.946 | 0.925 | 0.923 | 0.953 | 0.961 | - |
