# Supplementary material for: Different types of cultured human adult Cardiac Progenitor Cells have a high degree of transcriptome similarity
Source: J Cell Mol Med. 2014 Oct 14;18(11):2147–51. doi: 10.1111/jcmm.12458 (PMC4224548; doi:10.1111/jcmm.12458)
Supplement: Table S3 — (a) Significantly Differentially expressed genes between monolayer growing CPCs groups. (b) Significantly differentially expressed genes between CSps and monolayer growing CPCs. [file jcmm0018-2147-sd5.doc]

**Supplementary Table 3a**: Monolayer growing CPCs comparison.

|  | **Comparison** | **Description** | **N. of Sign.**  **genes** |
| --- | --- | --- | --- |
| **Sca-1+ cells comparison** | Sca GEL SP++ (Avg) vs  Sca-CDCs GEL SP++ (Avg) | Genes up in Sca GEL SP++ | 0 |
|  |  | Genes up in Sca-CDCs GEL SP++ | 0 |
|  | Sca-CDCs GEL SP++ (Avg) vs Sca-CDCs FN CEM (Avg) | Genes up in Sca-CDCs GEL SP++ | 0 |
|  |  | Genes up in Sca-CDCs FN CEM | 0 |
| **c-Kit+ cells comparison** | Kit-CDCs GEL SP++ (Avg) vs Kit-CDCs FN CEM (Avg) | Genes up in Kit-CDCs GEL SP++ | 0 |
|  |  | Genes up in Kit-CDCs FN CEM | 0 |
|  | Kit-CDCs GEL SP++ (Avg) vs Kit GEL SP++ (Avg) | Genes up in Kit-CDCs GEL SP++ | 0 |
|  |  | Genes up in Kit GEL SP++ | 0 |
|  | Kit-CDCs GEL SP++ (Avg) vs Kit K-Med (Avg) | Genes up in Kit-CDCs GEL SP++ | 1 |
|  |  | Genes up in Kit K-Med | 0 |
|  | Kit K-Med (Avg)-  Kit-CDCs FN CEM (Avg) | Genes up in Kit K-Med | 0 |
|  |  | Genes up in Kit-CDCs FN CEM | 0 |
|  | Kit GEL SP++ (Avg)-  Kit K-Med (Avg) | Genes up in Kit GEL SP++ | 0 |
|  |  | Genes up in Kit K-Med | 1 |
| **c-Kit+ vs Sca-1+ cells comparison** | Sca GEL SP++ (Avg) vs  Kit-CDCs GEL SP++ | Genes up in Sca GEL SP++ | 1 |
|  |  | Genes up in Kit-CDCs GEL SP++ | 1 |
|  | Sca GEL SP++ (Avg) vs  Kit GEL SP++ (Avg) | Genes up in Sca GEL SP++ | 0 |
|  |  | Genes up in Kit GEL SP++ | 0 |
|  | Sca GEL SP++ (Avg)-  Kit K-Med (Avg) | Genes up in Sca GEL SP++ | 1 |
|  |  | Genes up in Kit K-Med | 3 |
|  | Kit-CDCs GEL SP++ (Avg) vs Sca-CDCs GEL SP++ (Avg) | Genes up in Kit-CDCs GEL SP++ | 0 |
|  |  | Genes up in Sca-CDCs GEL SP++ | 0 |
|  | Kit-CDCs FN CEM (Avg) vs Sca-CDCs FN CEM (Avg) | Genes up in Kit-CDCs FN CEM | 0 |
|  |  | Genes up in Sca-CDCs FN CEM | 0 |
| **Sca-1/ c-kit vs CDCs**  **comparison** | Sca GEL SP++ (Avg)-  CDCs GEL SP++ (Avg) | Genes up in Sca GEL SP++ | 4 |
|  |  | Genes up in CDCs GEL SP++ | 11 |
|  | Sca-CDCs GEL SP++ (Avg)-  CDCs GEL SP++ (Avg) | Genes up in Sca-CDCs GEL SP++ | 0 |
|  |  | Genes up in CDCs GEL SP++ | 0 |
|  | Sca-CDCs FN CEM (Avg)-  CDCs FN CEM (Avg) | Genes up in Sca-CDCs FN CEM | 0 |
|  |  | Genes up in CDCs FN CEM | 0 |
|  | Kit-CDCs GEL SP++ (Avg)-  CDCs GEL SP++ (Avg) | Genes up in Kit-CDCs GEL SP++ | 4 |
|  |  | Genes up in CDCs GEL SP++ | 0 |
|  | Kit-CDCs FN CEM (Avg)-  CDCs FN CEM (Avg) | Genes up in Kit-CDCs FN CEM | 0 |
|  |  | Genes up in CDCs FN CEM | 0 |
|  | Kit GEL SP++ (Avg)-  CDCs GEL SP++ (Avg) | Genes up in Kit GEL SP++ | 0 |
|  |  | Genes up in CDCs GEL SP++ | 1 |
|  | Kit K-Med (Avg)-  CDCs FN CEM (Avg) | Genes up in Kit K-Med | 0 |
|  |  | Genes up in CDCs FN CEM | 0 |
| **CDCs comparison** | CDCs GEL SP++ (Avg)-  CDCs FN CEM (Avg) | Genes up in CDCs GEL SP++ | 0 |
|  |  | Genes up in CDCs FN CEM | 0 |

**Supplementary table 3b:** CSps comparison.

|  | **Comparison** | **Description** | **N. of Significant**  **genes** | **% total** |
| --- | --- | --- | --- | --- |
| **Sca-1 vs CSps**  **comparison** | Sca GEL SP++ (Avg)-  CSps (Avg) | Genes up in Sca GEL SP++ | 1,479 | 11.31426 |
|  |  | Genes up in CSps | 1,666 | 12.7448 |
|  | Sca-CDCs GEL SP++ (Avg)-  CSps (Avg) | Genes up in Sca-CDCs GEL SP++ | 1,052 | 8.047736 |
|  |  | Genes up in CSps | 1,151 | 8.80508 |
|  | Sca-CDCs FN CEM (Avg)-  CSps (Avg) | Genes up in Sca-CDCs FN CEM | 1,326 | 10.14382 |
|  |  | Genes up in CSps | 1,330 | 10.17442 |
| **c-Kit vs CSps**  **comparison** | Kit-CDCs GEL SP++ (Avg)-  CSps (Avg) | Genes up in Kit-CDCs GEL SP++ | 1,427 | 10.91646 |
|  |  | Genes up in CSps | 1,442 | 11.03121 |
|  | Kit-CDCs FN CEM (Avg)-  CSps (Avg) | Genes up in Kit-CDCs FN CEM | 1,190 | 9.103427 |
|  |  | Genes up in CSps | 1,373 | 10.50337 |
|  | Kit GEL SP++ (Avg)-  CSps (Avg) | Genes up in Kit GEL SP++ | 1,050 | 8.032436 |
|  |  | Genes up in CSps | 1,317 | 10.07497 |
|  | Kit K-Med (Avg)-  CSps (Avg) | Genes up in Kit K-Med | 88 | 0.673195 |
|  |  | Genes up in CSps | 154 | 1.178091 |
| **CDCs vs CSps comparison** | CDCs FN CEM (Avg)-  CSps (Avg) | Genes up in CDCs FN CEM | 159 | 1.21634 |
|  |  | Genes up in CSps | 332 | 2.53978 |
