# Supplementary material for: Different types of cultured human adult Cardiac Progenitor Cells have a high degree of transcriptome similarity
Source: J Cell Mol Med. 2014 Oct 14;18(11):2147–51. doi: 10.1111/jcmm.12458 (PMC4224548; doi:10.1111/jcmm.12458)
Supplement: Table S4 — List of significantly differentially expressed genes between monolayer growing CPCs. [file jcmm0018-2147-sd6.doc]

**Supplementary Table 4a.**

Genes upregulated in Sca GEL SP++ vs Kit K-Med.

|  |  |  |  |  |  |
| --- | --- | --- | --- | --- | --- |
| **Symbol** | **Entrez Gene Name** | **Log Ratio** | **p-value** | **Location** | **Function** |
| B3GNT5 | UDP-GlcNAc:betaGal beta-1,3-N-acetylglucosaminyltransferase 5 | 2.721 | 4.35E-02 | Cytoplasm | enzyme |

Genes upregulated in Kit K-Med vs Sca GEL SP++.

| **Symbol** | **Entrez Gene Name** | **Log Ratio** | **p-value** | **Location** | **Function** |
| --- | --- | --- | --- | --- | --- |
| CHAC1 | ChaC, cation transport regulator homolog 1 (E. coli) | 3.230 | 4.35E-02 | Cytoplasm | other |
| GPT2 | glutamic pyruvate transaminase (alanine aminotransferase) 2 | 2.800 | 4.40E-02 | Cytoplasm | enzyme |
| VLDLR | very low density lipoprotein receptor | 3.320 | 4.30E-02 | Plasma Membrane | transporter |

**Supplementary Table 4b.**

Genes up upregulated in Sca GEL SP++ vs CDCs GEL SP++ (Avg).

| **Symbol** | **Entrez Gene Name** | **Log Ratio** | **p-value** | **Location** | **Function** |
| --- | --- | --- | --- | --- | --- |
| PTMA | prothymosin, alpha | 2.320 | 3.00E-02 | Nucleus | other |
| SNRPE | small nuclear ribonucleoprotein polypeptide E | 2.047 | 3.61E-02 | Nucleus | other |
| HSPCAL3 | heat shock 90kDa protein 1, alpha-like 3 | 2.764 | 0.009 |  |  |
| LOC643308 |  | 2.880 | 0.117 | - | - |
| LOC401717 |  | 2.113 | 0.026 |  |  |
| LOC644380 |  | 2.427 | 0.036 |  |  |
| LOC645086 |  | 2.034 | 0.026 |  |  |
| LOC646784 |  | 2.208 | 0.036 |  |  |
| LOC400652 |  | 2.229 | 0.037 |  |  |
| LOC646786 |  | 2.104 | 0.037 |  |  |
| LOC100127893 |  | 2.102 | 0.041 |  |  |

Genes up upregulated in CDCs GEL SP++ vs Sca GEL SP++ (Avg).

| **Symbol** | **Entrez Gene Name** | **Log Ratio** | **p-value** | **Location** | **Function** |
| --- | --- | --- | --- | --- | --- |
| OAS2 | 2'-5'-oligoadenylate synthetase 2, 69/71kDa | 3.045 | 9.70E-03 | Cytoplasm | enzyme |
| NKD2 | naked cuticle homolog 2 (Drosophila) | 4.011 | 3.40E-02 | Nucleus | other |
| LOC100132761 |  | 3.980 | 0.034 |  |  |
| LOC100130332 |  | 2.170 | 0.038 |  |  |

**Supplementary Table 4c**

**Genes up upregulated in Kit-CDCs GEL SP++ vs CDCs GEL SP++**

| **Symbol** | **Entrez Gene Name** | **Log Ratio** | **p-value** | **Location** | **Function** |
| --- | --- | --- | --- | --- | --- |
| CXCL12 | chemokine (C-X-C motif) ligand 12 | 2.537 | 4.60E-02 | Extracellular Space | cytokine |
| LOC401717 |  | 2.540 | 0.046 |  |  |
| LOC649801 |  | 2.070 | 0.046 |  |  |
| LOC400652 |  | 2.570 | 0.046 |  |  |

**Supplementary Table 4d**

Genes up upregulated in Kit-CDCs GEL SP++ (Avg) vs Kit K-Med (Avg)

| **Symbol** | **Entrez Gene Name** | **Log Ratio** | **p-value** | **Location** | **Function** |
| --- | --- | --- | --- | --- | --- |
| CXCL12 | chemokine (C-X-C motif) ligand 12 | 4.126 | 1.60E-02 | Extracellular Space | cytokine |

**Supplementary Table 4e**

Genes up upregulated in Kit K-Med (Avg) vs Kit GEL SP++ (Avg)-

| **Symbol** | **Entrez Gene Name** | **Log Ratio** | **p-value** | **Location** | **Function** |
| --- | --- | --- | --- | --- | --- |
| VLDLR | very low density lipoprotein receptor | 3.588 | 6.00E-03 | Plasma Membrane | transporter |
