# Supplementary material for: Different types of cultured human adult Cardiac Progenitor Cells have a high degree of transcriptome similarity
Source: J Cell Mol Med. 2014 Oct 14;18(11):2147–51. doi: 10.1111/jcmm.12458 (PMC4224548; doi:10.1111/jcmm.12458)
Supplement: Table S5 — (a) List of significantly differentially expressed genes between CSps and Sca GEL SP++. (b) List of significantly differentially expressed genes between CSps and CDCs FN CEM. (c) List of significantly differentially expressed genes between CSps and Kit K-MED. [file jcmm0018-2147-sd7.doc]

**Supplementary Table 5a**

Genes upregulated in CDCs vs CSps

| **Symbol** | **Entrez Gene Name** | **Log Ratio** | **p-value** | **Location** | **Function** |
| --- | --- | --- | --- | --- | --- |
| NPPB | natriuretic peptide B | 6.613 | 2.75E-02 | Extracellular Space | Other |
| ANKRD1 | ankyrin repeat domain 1 (cardiac muscle) | 4.561 | 9.98E-03 | Cytoplasm | transcription regulator |
| ABI3BP | ABI family, member 3 (NESH) binding protein | 4.449 | 3.31E-02 | Extracellular Space | other |
| CPA4 | carboxypeptidase A4 | 4.361 | 1.46E-02 | Extracellular Space | peptidase |
| CLDN11 | claudin 11 | 3.191 | 1.08E-02 | Plasma Membrane | other |
| KRT7 | keratin 7 | 3.103 | 3.29E-02 | Cytoplasm | other |
| TAGLN | transgelin | 2.897 | 6.79E-03 | Cytoplasm | other |
| AHNAK2 | AHNAK nucleoprotein 2 | 2.674 | 1.96E-02 | Other | other |
| DDAH1 | dimethylarginine dimethylaminohydrolase 1 | 2.558 | 1.87E-02 | Cytoplasm | enzyme |
| LOC645638 | WDNM1-like pseudogene | 2.553 | 1.57E-02 | Other | other |
| MYL9 | myosin, light chain 9, regulatory | 2.49 | 2.05E-02 | Cytoplasm | other |
| MGARP | mitochondria-localized glutamic acid-rich protein | 2.472 | 6.89E-03 | Cytoplasm | other |
| TUFT1 | tuftelin 1 | 2.407 | 1.98E-02 | Other | other |
| STC2 | stanniocalcin 2 | 2.376 | 4.15E-03 | Extracellular Space | other |
| OXTR | oxytocin receptor | 2.364 | 2.30E-02 | Plasma Membrane | G-protein coupled receptor |
| LACTB | lactamase, beta | 2.313 | 1.45E-02 | Cytoplasm | other |
| CRIM1 | cysteine rich transmembrane BMP regulator 1 (chordin-like) | 2.273 | 2.24E-02 | Extracellular Space | kinase |
| HAPLN1 | hyaluronan and proteoglycan link protein 1 | 2.197 | 1.83E-02 | Extracellular Space | other |
| BCYRN1 | brain cytoplasmic RNA 1 | 2.194 | 4.13E-03 | Other | other |
| FRMD6 | FERM domain containing 6 | 2.19 | 7.77E-03 | Cytoplasm | other |
| SH3RF2 | SH3 domain containing ring finger 2 | 2.18 | 2.44E-02 | Extracellular Space | other |
| GBP1 | guanylate binding protein 1, interferon-inducible | 2.149 | 3.56E-02 | Cytoplasm | enzyme |
| PTPLA | protein tyrosine phosphatase-like (proline instead of catalytic arginine), member A | 2.138 | 1.98E-02 | Other | phosphatase |
| SYNC | syncoilin, intermediate filament protein | 2.125 | 2.87E-02 | Cytoplasm | other |
| TGFB2 | transforming growth factor, beta 2 | 2.123 | 3.45E-02 | Extracellular Space | growth factor |
| THBS1 | thrombospondin 1 | 2.117 | 2.33E-02 | Extracellular Space | other |
| CDC42EP3 | CDC42 effector protein (Rho GTPase binding) 3 | 2.106 | 2.16E-02 | Cytoplasm | other |
| C7orf10 | chromosome 7 open reading frame 10 | 2.104 | 3.53E-02 | Cytoplasm | other |
| EPDR1 | ependymin related protein 1 (zebrafish) | 2.073 | 1.45E-02 | Nucleus | other |
| GAS6 | growth arrest-specific 6 | 2.048 | 4.94E-02 | Extracellular Space | growth factor |
| C10orf76 | chromosome 10 open reading frame 76 | 2.045 | 7.77E-03 | Other | other |
| ALDH1B1 | aldehyde dehydrogenase 1 family, member B1 | 2.044 | 2.97E-02 | Cytoplasm | enzyme |
| BST1 | bone marrow stromal cell antigen 1 | 2.014 | 4.70E-02 | Plasma Membrane | enzyme |
| GADD45A | growth arrest and DNA-damage-inducible, alpha | 2.01 | 1.46E-02 | Nucleus | other |

**Genes upregulated in CSps vs CDCs**

| **Symbol** | **Entrez Gene Name** | **Log Ratio** | **p-value** | **Location** | **Function** |
| --- | --- | --- | --- | --- | --- |
| MGP | matrix Gla protein | 5.909 | 4.46E-03 | Extracellular Space | other |
| APOE | apolipoprotein E | 5.65 | 2.78E-03 | Extracellular Space | transporter |
| CCL7 | chemokine (C-C motif) ligand 7 | 5.261 | 1.92E-03 | Extracellular Space | cytokine |
| IL11 | interleukin 11 | 4.991 | 2.47E-04 | Extracellular Space | cytokine |
| EGR2 | early growth response 2 | 4.794 | 3.87E-03 | Nucleus | transcription regulator |
| RASD1 | RAS, dexamethasone-induced 1 | 4.764 | 5.70E-03 | Cytoplasm | enzyme |
| CRLF1 | cytokine receptor-like factor 1 | 4.508 | 2.20E-03 | Extracellular Space | other |
| TGFB3 | transforming growth factor, beta 3 | 4.436 | 2.71E-03 | Extracellular Space | growth factor |
| HSD11B1 | hydroxysteroid (11-beta) dehydrogenase 1 | 4.215 | 1.89E-02 | Cytoplasm | enzyme |
| NR4A2 | nuclear receptor subfamily 4, group A, member 2 | 4.183 | 3.87E-03 | Nucleus | ligand-dependent nuclear receptor |
| RGCC | regulator of cell cycle | 3.96 | 2.16E-02 | Cytoplasm | other |
| TNC | tenascin C | 3.858 | 8.49E-03 | Extracellular Space | other |
| MXRA5 | matrix-remodelling associated 5 | 3.849 | 2.16E-02 | Extracellular Space | other |
| MMP10 | matrix metallopeptidase 10 (stromelysin 2) | 3.806 | 8.16E-03 | Extracellular Space | peptidase |
| COL7A1 | collagen, type VII, alpha 1 | 3.635 | 3.32E-03 | Extracellular Space | other |
| NDP | Norrie disease (pseudoglioma) | 3.617 | 4.13E-03 | Extracellular Space | growth factor |
| GAS1 | growth arrest-specific 1 | 3.559 | 1.89E-02 | Plasma Membrane | other |
| CCL8 | chemokine (C-C motif) ligand 8 | 3.512 | 2.90E-02 | Extracellular Space | cytokine |
| PTPRN | protein tyrosine phosphatase, receptor type, N | 3.482 | 6.37E-04 | Plasma Membrane | phosphatase |
| VASH1 | vasohibin 1 | 3.468 | 2.76E-03 | Extracellular Space | other |
| ADAMTSL2 | ADAMTS-like 2 | 3.392 | 2.78E-02 | Other | other |
| GPX3 | glutathione peroxidase 3 (plasma) | 3.354 | 7.89E-04 | Extracellular Space | enzyme |
| TACSTD2 | tumor-associated calcium signal transducer 2 | 3.316 | 2.16E-02 | Plasma Membrane | other |
| PTGES | prostaglandin E synthase | 3.314 | 1.45E-02 | Cytoplasm | enzyme |
| TMEM59L | transmembrane protein 59-like | 3.311 | 4.19E-03 | Cytoplasm | other |
| ISG20 | interferon stimulated exonuclease gene 20kDa | 3.277 | 4.15E-03 | Nucleus | enzyme |
| MFAP4 | microfibrillar-associated protein 4 | 3.267 | 3.87E-03 | Extracellular Space | other |
| RAMP1 | receptor (G protein-coupled) activity modifying protein 1 | 3.229 | 4.03E-03 | Plasma Membrane | transporter |
| SLC6A9 | solute carrier family 6 (neurotransmitter transporter, glycine), member 9 | 3.197 | 4.03E-03 | Plasma Membrane | transporter |
| IFITM1 | interferon induced transmembrane protein 1 | 3.181 | 1.89E-02 | Plasma Membrane | transmembrane receptor |
| CALCRL | calcitonin receptor-like | 3.117 | 5.35E-03 | Plasma Membrane | G-protein coupled receptor |
| SMOX | spermine oxidase | 3.071 | 4.15E-03 | Cytoplasm | enzyme |
| PDGFRB | platelet-derived growth factor receptor, beta polypeptide | 3.031 | 4.73E-03 | Plasma Membrane | kinase |
| PAPLN | papilin, proteoglycan-like sulfated glycoprotein | 3.031 | 3.58E-03 | Extracellular Space | other |
| FGL2 | fibrinogen-like 2 | 3 | 1.76E-02 | Extracellular Space | peptidase |
| ISLR | immunoglobulin superfamily containing leucine-rich repeat | 2.992 | 4.11E-03 | Extracellular Space | other |
| NPTX2 | neuronal pentraxin II | 2.99 | 2.16E-02 | Extracellular Space | other |
| TNFRSF19 | tumor necrosis factor receptor superfamily, member 19 | 2.988 | 4.13E-03 | Plasma Membrane | transmembrane receptor |
| PDK4 | pyruvate dehydrogenase kinase, isozyme 4 | 2.93 | 4.11E-03 | Cytoplasm | kinase |
| RDH10 | retinol dehydrogenase 10 (all-trans) | 2.886 | 1.98E-02 | Nucleus | enzyme |
| IGFBP1 | insulin-like growth factor binding protein 1 | 2.866 | 3.05E-03 | Extracellular Space | other |
| C2CD4B | C2 calcium-dependent domain containing 4B | 2.835 | 6.34E-03 | Other | other |
| DCN | decorin | 2.814 | 2.72E-02 | Extracellular Space | other |
| PLCB1 | phospholipase C, beta 1 (phosphoinositide-specific) | 2.81 | 4.15E-03 | Cytoplasm | enzyme |
| PTGS2 | prostaglandin-endoperoxide synthase 2 (prostaglandin G/H synthase and cyclooxygenase) | 2.747 | 3.87E-03 | Cytoplasm | enzyme |
| GABBR2 | gamma-aminobutyric acid (GABA) B receptor, 2 | 2.733 | 4.51E-02 | Plasma Membrane | G-protein coupled receptor |
| CSGALNACT1 | chondroitin sulfate N-acetylgalactosaminyltransferase 1 | 2.723 | 1.57E-02 | Cytoplasm | enzyme |
| DUSP5 | dual specificity phosphatase 5 | 2.671 | 4.11E-03 | Nucleus | phosphatase |
| ANGPTL2 | angiopoietin-like 2 | 2.649 | 2.44E-02 | Extracellular Space | other |
| IL33 | interleukin 33 | 2.633 | 1.25E-02 | Extracellular Space | cytokine |
| ADCY4 | adenylate cyclase 4 | 2.599 | 4.72E-03 | Plasma Membrane | enzyme |
| RAP1GAP | RAP1 GTPase activating protein | 2.578 | 9.98E-03 | Cytoplasm | other |
| MST1 | macrophage stimulating 1 (hepatocyte growth factor-like) | 2.572 | 4.11E-03 | Extracellular Space | growth factor |
| IL7R | interleukin 7 receptor | 2.562 | 8.33E-03 | Plasma Membrane | transmembrane receptor |
| BMP2 | bone morphogenetic protein 2 | 2.562 | 8.33E-03 | Extracellular Space | growth factor |
| BTG1 | B-cell translocation gene 1, anti-proliferative | 2.518 | 3.58E-03 | Nucleus | transcription regulator |
| FAM46A | family with sequence similarity 46, member A | 2.517 | 2.55E-02 | Other | other |
| PDE4B | phosphodiesterase 4B, cAMP-specific | 2.508 | 1.89E-02 | Cytoplasm | enzyme |
| EDNRB | endothelin receptor type B | 2.505 | 4.15E-03 | Plasma Membrane | G-protein coupled receptor |
| SOBP | sine oculis binding protein homolog (Drosophila) | 2.496 | 4.15E-03 | Nucleus | other |
| HGF | hepatocyte growth factor (hepapoietin A; scatter factor) | 2.486 | 2.22E-02 | Extracellular Space | growth factor |
| CFB | complement factor B | 2.475 | 2.04E-02 | Extracellular Space | peptidase |
| LOC339192 | uncharacterized LOC339192 | 2.455 | 1.44E-02 | Other | other |
| SOX9 | SRY (sex determining region Y)-box 9 | 2.454 | 1.19E-02 | Nucleus | transcription regulator |
| SPRY1 | sprouty homolog 1, antagonist of FGF signaling (Drosophila) | 2.452 | 1.21E-02 | Other | other |
| TREM1 | triggering receptor expressed on myeloid cells 1 | 2.446 | 1.89E-02 | Plasma Membrane | transmembrane receptor |
| HEY1 | hairy/enhancer-of-split related with YRPW motif 1 | 2.426 | 1.51E-02 | Nucleus | transcription regulator |
| CYP26B1 | cytochrome P450, family 26, subfamily B, polypeptide 1 | 2.409 | 2.69E-02 | Cytoplasm | enzyme |
| CHI3L2 | chitinase 3-like 2 | 2.397 | 5.40E-03 | Extracellular Space | enzyme |
| PARPBP | PARP1 binding protein | 2.379 | 1.98E-02 | Nucleus | other |
| HTRA1 | HtrA serine peptidase 1 | 2.34 | 1.24E-02 | Extracellular Space | peptidase |
| SERPINF1 | serpin peptidase inhibitor, clade F (alpha-2 antiplasmin, pigment epithelium derived factor), member 1 | 2.34 | 2.44E-02 | Extracellular Space | other |
| SPTSSA | serine palmitoyltransferase, small subunit A | 2.335 | 3.87E-03 | Cytoplasm | enzyme |
| COL18A1 | collagen, type XVIII, alpha 1 | 2.331 | 3.46E-03 | Extracellular Space | other |
| LIF | leukemia inhibitory factor | 2.319 | 2.23E-02 | Extracellular Space | cytokine |
| MINOS1-NBL1/NBL1 | neuroblastoma 1, DAN family BMP antagonist | 2.308 | 4.13E-03 | Nucleus | other |
| LAMA5 | laminin, alpha 5 | 2.28 | 4.85E-02 | Extracellular Space | other |
| PTGER2 | prostaglandin E receptor 2 (subtype EP2), 53kDa | 2.277 | 2.85E-02 | Plasma Membrane | G-protein coupled receptor |
| LRFN5 | leucine rich repeat and fibronectin type III domain containing 5 | 2.273 | 4.63E-03 | Nucleus | other |
| SLC22A17 | solute carrier family 22, member 17 | 2.252 | 1.98E-02 | Plasma Membrane | transporter |
| SHC4 | SHC (Src homology 2 domain containing) family, member 4 | 2.241 | 2.07E-02 | Cytoplasm | other |
| MEIS1 | Meis homeobox 1 | 2.238 | 2.16E-02 | Nucleus | transcription regulator |
| PLAT | plasminogen activator, tissue | 2.228 | 1.25E-02 | Extracellular Space | peptidase |
| BDKRB2 | bradykinin receptor B2 | 2.215 | 4.94E-02 | Plasma Membrane | G-protein coupled receptor |
| SERPIND1 | serpin peptidase inhibitor, clade D (heparin cofactor), member 1 | 2.188 | 1.34E-02 | Extracellular Space | other |
| TMEM178A | transmembrane protein 178A | 2.183 | 9.98E-03 | Other | other |
| APOD | apolipoprotein D | 2.18 | 1.25E-02 | Extracellular Space | transporter |
| NOTCH3 | notch 3 | 2.15 | 3.37E-02 | Plasma Membrane | transcription regulator |
| ITGA10 | integrin, alpha 10 | 2.138 | 6.89E-03 | Plasma Membrane | other |
| CCDC151 | coiled-coil domain containing 151 | 2.119 | 3.25E-02 | Other | other |
| GSTM3 | glutathione S-transferase mu 3 (brain) | 2.109 | 2.07E-02 | Cytoplasm | enzyme |
| PDGFD | platelet derived growth factor D | 2.105 | 4.98E-02 | Extracellular Space | growth factor |
| TMX4 | thioredoxin-related transmembrane protein 4 | 2.104 | 4.15E-03 | Cytoplasm | enzyme |
| C14orf132 | chromosome 14 open reading frame 132 | 2.092 | 1.76E-02 | Other | other |
| FMNL3 | formin-like 3 | 2.082 | 4.19E-03 | Cytoplasm | other |
| TTYH2 | tweety homolog 2 (Drosophila) | 2.056 | 2.64E-02 | Other | ion channel |
| CD93 | CD93 molecule | 2.049 | 2.07E-02 | Plasma Membrane | other |
| PECAM1 | platelet/endothelial cell adhesion molecule 1 | 2.049 | 4.62E-02 | Plasma Membrane | other |
| PRSS3 | protease, serine, 3 | 2.005 | 2.22E-02 | Extracellular Space | peptidase |

**Supplementary Table 5b**

Genes up regulated in Sca vs CSps

| **Symbol** | **Entrez Gene Name** | **Log Ratio** | **p-value** | **Location** | **Type(s)** |
| --- | --- | --- | --- | --- | --- |
| LOC644936 | actin, beta pseudogene | 4.843 | 2.81E-03 | Other | other |
| MAOA | monoamine oxidase A | 3.428 | 8.77E-03 | Cytoplasm | enzyme |
| ABI3BP | ABI family, member 3 (NESH) binding protein | 3.427 | 1.50E-02 | Extracellular Space | other |
| ANKRD1 | ankyrin repeat domain 1 (cardiac muscle) | 3.338 | 9.89E-03 | Cytoplasm | transcription regulator |
| KRT7 | keratin 7 | 3.305 | 9.23E-03 | Cytoplasm | other |
| EFNB2 | ephrin-B2 | 3.275 | 1.77E-02 | Plasma Membrane | other |
| CPA4 | carboxypeptidase A4 | 3.194 | 3.78E-02 | Extracellular Space | peptidase |
| CLDN1 | claudin 1 | 3.192 | 4.27E-02 | Plasma Membrane | other |
| SORBS2 | sorbin and SH3 domain containing 2 | 2.838 | 1.62E-02 | Plasma Membrane | other |
| CRIM1 | cysteine rich transmembrane BMP regulator 1 (chordin-like) | 2.834 | 8.88E-04 | Extracellular Space | kinase |
| SERPINB2 | serpin peptidase inhibitor, clade B (ovalbumin), member 2 | 2.822 | 2.03E-02 | Extracellular Space | other |
| SFTA1P | surfactant associated 1, pseudogene | 2.821 | 2.11E-03 | Other | other |
| NEXN | nexilin (F actin binding protein) | 2.812 | 1.17E-03 | Plasma Membrane | other |
| BCYRN1 | brain cytoplasmic RNA 1 | 2.689 | 8.88E-04 | Other | other |
| DNAJB4 | DnaJ (Hsp40) homolog, subfamily B, member 4 | 2.688 | 8.88E-04 | Nucleus | other |
| AMY1A (includes others) | amylase, alpha 1A (salivary) | 2.598 | 3.90E-03 | Extracellular Space | enzyme |
| SNORD80 | small nucleolar RNA, C/D box 80 | 2.564 | 1.34E-03 | Other | other |
| BCHE | butyrylcholinesterase | 2.546 | 1.71E-02 | Plasma Membrane | enzyme |
| CDC42EP3 | CDC42 effector protein (Rho GTPase binding) 3 | 2.473 | 3.52E-03 | Cytoplasm | other |
| TUBA3C/TUBA3D | tubulin, alpha 3c | 2.413 | 2.48E-03 | Other | other |
| RUNX2 | runt-related transcription factor 2 | 2.387 | 5.98E-03 | Nucleus | transcription regulator |
| NUF2 | NUF2, NDC80 kinetochore complex component | 2.374 | 1.48E-02 | Nucleus | other |
| DLC1 | deleted in liver cancer 1 | 2.367 | 1.84E-02 | Cytoplasm | other |
| PTMA | prothymosin, alpha | 2.324 | 1.49E-03 | Nucleus | other |
| RNF144B | ring finger protein 144B | 2.32 | 6.57E-03 | Other | enzyme |
| SUMO1 | small ubiquitin-like modifier 1 | 2.293 | 2.21E-03 | Nucleus | enzyme |
| CAPZA1 | capping protein (actin filament) muscle Z-line, alpha 1 | 2.289 | 6.56E-04 | Cytoplasm | other |
| THBS1 | thrombospondin 1 | 2.261 | 6.27E-03 | Extracellular Space | other |
| TMEM156 | transmembrane protein 156 | 2.243 | 2.41E-02 | Other | other |
| LOC728026 | prothymosin alpha-like | 2.241 | 3.34E-03 | Other | other |
| MBP | myelin basic protein | 2.205 | 9.13E-03 | Extracellular Space | other |
| UGCG | UDP-glucose ceramide glucosyltransferase | 2.181 | 1.94E-02 | Cytoplasm | enzyme |
| NAP1L1 | nucleosome assembly protein 1-like 1 | 2.17 | 1.86E-03 | Nucleus | other |
| SNRPE | small nuclear ribonucleoprotein polypeptide E | 2.16 | 1.10E-03 | Nucleus | other |
| LOC100288142/NBPF8 | neuroblastoma breakpoint family, member 8 | 2.156 | 1.49E-02 | Other | other |
| POLR3G | polymerase (RNA) III (DNA directed) polypeptide G (32kD) | 2.119 | 5.77E-03 | Nucleus | enzyme |
| TMEM154 | transmembrane protein 154 | 2.114 | 2.05E-02 | Other | other |
| ARHGAP18 | Rho GTPase activating protein 18 | 2.109 | 2.79E-03 | Cytoplasm | other |
| PKN2 | protein kinase N2 | 2.105 | 3.29E-03 | Cytoplasm | kinase |
| ROR1 | receptor tyrosine kinase-like orphan receptor 1 | 2.104 | 1.40E-02 | Plasma Membrane | kinase |
| ALDH1B1 | aldehyde dehydrogenase 1 family, member B1 | 2.087 | 1.49E-03 | Cytoplasm | enzyme |
| DNTTIP2 | deoxynucleotidyltransferase, terminal, interacting protein 2 | 2.079 | 2.27E-03 | Nucleus | other |
| SRSF3 | serine/arginine-rich splicing factor 3 | 2.075 | 4.15E-03 | Nucleus | other |
| HMGB2 | high mobility group box 2 | 2.07 | 1.82E-02 | Nucleus | transcription regulator |
| RASSF6 | Ras association (RalGDS/AF-6) domain family member 6 | 2.066 | 3.29E-03 | Other | other |
| PDGFC | platelet derived growth factor C | 2.04 | 3.44E-03 | Extracellular Space | growth factor |
| ACTR3 | ARP3 actin-related protein 3 homolog (yeast) | 2.036 | 1.89E-03 | Plasma Membrane | other |
| RNF144A | ring finger protein 144A | 2.027 | 1.68E-02 | Nucleus | other |
| MFI2 | antigen p97 (melanoma associated) identified by monoclonal antibodies 133.2 and 96.5 | 2.008 | 3.35E-03 | Plasma Membrane | other |
| ANP32A | acidic (leucine-rich) nuclear phosphoprotein 32 family, member A | 2.005 | 1.23E-03 | Nucleus | other |
| KIAA1033 | KIAA1033 | 2.005 | 7.58E-03 | Cytoplasm | other |

Genes up regulated in CSps vs Sca GEL SP++

| **Symbol** | **Entrez Gene Name** | **Log Ratio** | **p-value** | **Location** | **Type(s)** |
| --- | --- | --- | --- | --- | --- |
| RARRES2 | retinoic acid receptor responder (tazarotene induced) 2 | 6.787 | 1.04E-03 | Plasma Membrane | transmembrane receptor |
| CST1 | cystatin SN | 6.136 | 2.52E-05 | Other | other |
| EGR2 | early growth response 2 | 6.026 | 1.23E-04 | Nucleus | transcription regulator |
| GPNMB | glycoprotein (transmembrane) nmb | 5.487 | 7.53E-05 | Plasma Membrane | enzyme |
| GPX3 | glutathione peroxidase 3 (plasma) | 5.355 | 2.25E-04 | Extracellular Space | enzyme |
| CCL7 | chemokine (C-C motif) ligand 7 | 5.242 | 9.69E-05 | Extracellular Space | cytokine |
| APOE | apolipoprotein E | 5.145 | 3.02E-03 | Extracellular Space | transporter |
| MGP | matrix Gla protein | 5.033 | 2.12E-03 | Extracellular Space | other |
| IL11 | interleukin 11 | 4.919 | 4.13E-03 | Extracellular Space | cytokine |
| KIAA1199 | KIAA1199 | 4.904 | 1.27E-02 | Cytoplasm | other |
| CCL8 | chemokine (C-C motif) ligand 8 | 4.8 | 1.81E-03 | Extracellular Space | cytokine |
| PAMR1 | peptidase domain containing associated with muscle regeneration 1 | 4.755 | 2.49E-03 | Extracellular Space | peptidase |
| APOA1 | apolipoprotein A-I | 4.749 | 5.41E-03 | Extracellular Space | transporter |
| TGFB3 | transforming growth factor, beta 3 | 4.74 | 2.49E-04 | Extracellular Space | growth factor |
| IL24 | interleukin 24 | 4.633 | 5.08E-03 | Extracellular Space | cytokine |
| TNC | tenascin C | 4.594 | 1.49E-03 | Extracellular Space | other |
| IL33 | interleukin 33 | 4.376 | 3.52E-03 | Extracellular Space | cytokine |
| CRLF1 | cytokine receptor-like factor 1 | 4.328 | 1.47E-03 | Extracellular Space | other |
| CTSK | cathepsin K | 4.315 | 9.69E-03 | Cytoplasm | peptidase |
| NR4A2 | nuclear receptor subfamily 4, group A, member 2 | 4.301 | 2.04E-04 | Nucleus | ligand-dependent nuclear receptor |
| MEDAG | mesenteric estrogen-dependent adipogenesis | 4.167 | 3.75E-03 | Cytoplasm | other |
| IFITM1 | interferon induced transmembrane protein 1 | 4.135 | 2.12E-03 | Plasma Membrane | transmembrane receptor |
| ISG20 | interferon stimulated exonuclease gene 20kDa | 4.015 | 1.38E-03 | Nucleus | enzyme |
| LIF | leukemia inhibitory factor | 3.976 | 2.28E-03 | Extracellular Space | cytokine |
| TNFRSF11B | tumor necrosis factor receptor superfamily, member 11b | 3.973 | 1.83E-02 | Plasma Membrane | transmembrane receptor |
| MXRA5 | matrix-remodelling associated 5 | 3.945 | 3.16E-02 | Extracellular Space | other |
| ADIRF | adipogenesis regulatory factor | 3.891 | 2.56E-03 | Nucleus | other |
| NKD2 | naked cuticle homolog 2 (Drosophila) | 3.882 | 2.93E-03 | Nucleus | other |
| RDH10 | retinol dehydrogenase 10 (all-trans) | 3.869 | 1.47E-03 | Nucleus | enzyme |
| TACSTD2 | tumor-associated calcium signal transducer 2 | 3.788 | 2.04E-04 | Plasma Membrane | other |
| TCF21 | transcription factor 21 | 3.759 | 8.88E-04 | Nucleus | transcription regulator |
| COL7A1 | collagen, type VII, alpha 1 | 3.754 | 2.12E-03 | Extracellular Space | other |
| ECM1 | extracellular matrix protein 1 | 3.75 | 2.04E-04 | Extracellular Space | transporter |
| HEPH | hephaestin | 3.702 | 8.96E-04 | Plasma Membrane | transporter |
| GPC4 | glypican 4 | 3.619 | 3.11E-03 | Plasma Membrane | transmembrane receptor |
| RASD1 | RAS, dexamethasone-induced 1 | 3.616 | 3.39E-02 | Cytoplasm | enzyme |
| VEGFA | vascular endothelial growth factor A | 3.547 | 6.56E-04 | Extracellular Space | growth factor |
| CPXM2 | carboxypeptidase X (M14 family), member 2 | 3.511 | 3.29E-04 | Extracellular Space | peptidase |
| C1R | complement component 1, r subcomponent | 3.504 | 4.90E-03 | Extracellular Space | peptidase |
| EMILIN1 | elastin microfibril interfacer 1 | 3.503 | 7.31E-04 | Extracellular Space | other |
| MMP10 | matrix metallopeptidase 10 (stromelysin 2) | 3.475 | 4.08E-03 | Extracellular Space | peptidase |
| FAM46A | family with sequence similarity 46, member A | 3.473 | 3.95E-03 | Other | other |
| FGL2 | fibrinogen-like 2 | 3.47 | 1.99E-03 | Extracellular Space | peptidase |
| ANGPTL2 | angiopoietin-like 2 | 3.47 | 8.88E-04 | Extracellular Space | other |
| CNIH3 | cornichon homolog 3 (Drosophila) | 3.407 | 3.97E-04 | Plasma Membrane | transporter |
| OAS2 | 2'-5'-oligoadenylate synthetase 2, 69/71kDa | 3.356 | 3.05E-04 | Cytoplasm | enzyme |
| PTPRN | protein tyrosine phosphatase, receptor type, N | 3.344 | 8.88E-04 | Plasma Membrane | phosphatase |
| PTGDS | prostaglandin D2 synthase 21kDa (brain) | 3.338 | 1.58E-03 | Cytoplasm | enzyme |
| ADAMTSL2 | ADAMTS-like 2 | 3.326 | 6.42E-03 | Other | other |
| ENO2 | enolase 2 (gamma, neuronal) | 3.318 | 2.39E-03 | Cytoplasm | enzyme |
| STC1 | stanniocalcin 1 | 3.299 | 1.28E-02 | Extracellular Space | kinase |
| VWCE | von Willebrand factor C and EGF domains | 3.297 | 8.96E-03 | Other | other |
| MSC | musculin | 3.277 | 4.86E-02 | Cytoplasm | transcription regulator |
| NDP | Norrie disease (pseudoglioma) | 3.268 | 2.27E-02 | Extracellular Space | growth factor |
| CXCR4 | chemokine (C-X-C motif) receptor 4 | 3.257 | 2.14E-02 | Plasma Membrane | G-protein coupled receptor |
| HSD11B1 | hydroxysteroid (11-beta) dehydrogenase 1 | 3.249 | 8.64E-03 | Cytoplasm | enzyme |
| PAPLN | papilin, proteoglycan-like sulfated glycoprotein | 3.24 | 3.05E-04 | Extracellular Space | other |
| C1S | complement component 1, s subcomponent | 3.24 | 4.48E-03 | Extracellular Space | peptidase |
| TMEM59L | transmembrane protein 59-like | 3.208 | 1.49E-03 | Cytoplasm | other |
| EDNRB | endothelin receptor type B | 3.2 | 2.39E-04 | Plasma Membrane | G-protein coupled receptor |
| C1orf54 | chromosome 1 open reading frame 54 | 3.197 | 1.47E-02 | Other | other |
| SMAD6 | SMAD family member 6 | 3.181 | 3.79E-03 | Nucleus | transcription regulator |
| SLC6A9 | solute carrier family 6 (neurotransmitter transporter, glycine), member 9 | 3.178 | 8.88E-04 | Plasma Membrane | transporter |
| S100A4 | S100 calcium binding protein A4 | 3.176 | 4.25E-02 | Cytoplasm | other |
| CH25H | cholesterol 25-hydroxylase | 3.163 | 6.56E-04 | Cytoplasm | enzyme |
| CLCF1 | cardiotrophin-like cytokine factor 1 | 3.162 | 3.97E-04 | Extracellular Space | cytokine |
| LMO2 | LIM domain only 2 (rhombotin-like 1) | 3.153 | 2.95E-03 | Nucleus | other |
| PLCB1 | phospholipase C, beta 1 (phosphoinositide-specific) | 3.142 | 2.10E-02 | Cytoplasm | enzyme |
| HSPA12A | heat shock 70kDa protein 12A | 3.111 | 7.76E-03 | Other | other |
| ZNF467 | zinc finger protein 467 | 3.108 | 3.01E-03 | Nucleus | other |
| PCOLCE | procollagen C-endopeptidase enhancer | 3.104 | 1.24E-02 | Extracellular Space | other |
| MX1 | myxovirus (influenza virus) resistance 1, interferon-inducible protein p78 (mouse) | 3.096 | 3.14E-02 | Cytoplasm | enzyme |
| PDGFRB | platelet-derived growth factor receptor, beta polypeptide | 3.083 | 8.56E-03 | Plasma Membrane | kinase |
| CFB | complement factor B | 3.075 | 1.28E-02 | Extracellular Space | peptidase |
| BMP2 | bone morphogenetic protein 2 | 3.06 | 4.71E-02 | Extracellular Space | growth factor |
| IGFBP1 | insulin-like growth factor binding protein 1 | 3.057 | 2.04E-04 | Extracellular Space | other |
| DPYSL4 | dihydropyrimidinase-like 4 | 3.038 | 4.11E-02 | Cytoplasm | enzyme |
| ISLR | immunoglobulin superfamily containing leucine-rich repeat | 3.026 | 7.31E-04 | Extracellular Space | other |
| RAMP1 | receptor (G protein-coupled) activity modifying protein 1 | 3.026 | 1.49E-03 | Plasma Membrane | transporter |
| SMOX | spermine oxidase | 3.018 | 3.43E-03 | Cytoplasm | enzyme |
| LDB2 | LIM domain binding 2 | 3.016 | 3.29E-03 | Nucleus | transcription regulator |
| VASH1 | vasohibin 1 | 3.013 | 6.59E-03 | Extracellular Space | other |
| CTSF | cathepsin F | 2.987 | 2.04E-04 | Cytoplasm | peptidase |
| ABCC3 | ATP-binding cassette, sub-family C (CFTR/MRP), member 3 | 2.969 | 3.94E-03 | Plasma Membrane | transporter |
| SLC22A17 | solute carrier family 22, member 17 | 2.967 | 2.49E-03 | Plasma Membrane | transporter |
| C14orf132 | chromosome 14 open reading frame 132 | 2.887 | 2.30E-03 | Other | other |
| FBLN1 | fibulin 1 | 2.873 | 1.02E-02 | Extracellular Space | other |
| IGFBP6 | insulin-like growth factor binding protein 6 | 2.858 | 2.12E-03 | Extracellular Space | other |
| EBF3 | early B-cell factor 3 | 2.843 | 1.86E-02 | Nucleus | other |
| MFAP4 | microfibrillar-associated protein 4 | 2.841 | 2.53E-03 | Extracellular Space | other |
| MMP2 | matrix metallopeptidase 2 (gelatinase A, 72kDa gelatinase, 72kDa type IV collagenase) | 2.838 | 2.21E-03 | Extracellular Space | peptidase |
| PKNOX2 | PBX/knotted 1 homeobox 2 | 2.829 | 8.31E-04 | Nucleus | other |
| LAMA2 | laminin, alpha 2 | 2.819 | 3.11E-02 | Extracellular Space | other |
| DACT3 | dishevelled-binding antagonist of beta-catenin 3 | 2.802 | 9.52E-04 | Other | other |
| FMNL3 | formin-like 3 | 2.791 | 1.09E-03 | Cytoplasm | other |
| MFAP5 | microfibrillar associated protein 5 | 2.767 | 4.67E-02 | Extracellular Space | other |
| TRPV2 | transient receptor potential cation channel, subfamily V, member 2 | 2.766 | 3.02E-02 | Plasma Membrane | ion channel |
| AKR1C4 | aldo-keto reductase family 1, member C4 | 2.749 | 3.06E-02 | Cytoplasm | enzyme |
| NPTX2 | neuronal pentraxin II | 2.749 | 5.76E-03 | Extracellular Space | other |
| RFTN2 | raftlin family member 2 | 2.741 | 6.56E-04 | Other | other |
| DDIT4L | DNA-damage-inducible transcript 4-like | 2.736 | 1.61E-02 | Other | other |
| RAP1GAP | RAP1 GTPase activating protein | 2.727 | 1.61E-03 | Cytoplasm | other |
| ADCY4 | adenylate cyclase 4 | 2.723 | 1.49E-03 | Plasma Membrane | enzyme |
| CA9 | carbonic anhydrase IX | 2.721 | 1.28E-03 | Nucleus | enzyme |
| BTBD11 | BTB (POZ) domain containing 11 | 2.714 | 6.03E-03 | Other | transcription regulator |
| CYTH2 | cytohesin 2 | 2.704 | 8.88E-04 | Cytoplasm | other |
| ANKRD33 | ankyrin repeat domain 33 | 2.699 | 3.02E-03 | Nucleus | transcription regulator |
| LRP1 | low density lipoprotein receptor-related protein 1 | 2.695 | 8.75E-03 | Plasma Membrane | transmembrane receptor |
| SOX9 | SRY (sex determining region Y)-box 9 | 2.694 | 5.81E-03 | Nucleus | transcription regulator |
| SEZ6L2 | seizure related 6 homolog (mouse)-like 2 | 2.677 | 2.27E-03 | Other | other |
| ID2 | inhibitor of DNA binding 2, dominant negative helix-loop-helix protein | 2.639 | 2.51E-02 | Nucleus | transcription regulator |
| TMEM140 | transmembrane protein 140 | 2.628 | 6.56E-04 | Other | other |
| RAB3IL1 | RAB3A interacting protein (rabin3)-like 1 | 2.611 | 6.56E-04 | Other | other |
| MINOS1-NBL1/NBL1 | neuroblastoma 1, DAN family BMP antagonist | 2.595 | 8.79E-03 | Nucleus | other |
| SPAG4 | sperm associated antigen 4 | 2.595 | 1.78E-03 | Cytoplasm | other |
| PLEKHA4 | pleckstrin homology domain containing, family A (phosphoinositide binding specific) member 4 | 2.587 | 8.88E-04 | Cytoplasm | other |
| SERPINE2 | serpin peptidase inhibitor, clade E (nexin, plasminogen activator inhibitor type 1), member 2 | 2.586 | 9.03E-03 | Extracellular Space | other |
| C2CD4B | C2 calcium-dependent domain containing 4B | 2.577 | 2.51E-03 | Other | other |
| PPFIBP2 | PTPRF interacting protein, binding protein 2 (liprin beta 2) | 2.575 | 1.06E-03 | Nucleus | phosphatase |
| PCOLCE2 | procollagen C-endopeptidase enhancer 2 | 2.572 | 2.39E-02 | Extracellular Space | other |
| ALDOC | aldolase C, fructose-bisphosphate | 2.554 | 2.12E-03 | Cytoplasm | enzyme |
| IFI6 | interferon, alpha-inducible protein 6 | 2.552 | 1.94E-03 | Cytoplasm | other |
| GAA | glucosidase, alpha; acid | 2.55 | 8.88E-04 | Cytoplasm | enzyme |
| COL6A2 | collagen, type VI, alpha 2 | 2.547 | 1.49E-03 | Extracellular Space | other |
| CPT1C | carnitine palmitoyltransferase 1C | 2.544 | 1.18E-02 | Cytoplasm | enzyme |
| MAP1A | microtubule-associated protein 1A | 2.543 | 6.57E-03 | Cytoplasm | other |
| CALCRL | calcitonin receptor-like | 2.528 | 1.92E-03 | Plasma Membrane | G-protein coupled receptor |
| FAP | fibroblast activation protein, alpha | 2.527 | 1.41E-02 | Cytoplasm | peptidase |
| RTKN | rhotekin | 2.516 | 3.43E-03 | Cytoplasm | other |
| HGD | homogentisate 1,2-dioxygenase | 2.512 | 2.77E-03 | Cytoplasm | enzyme |
| GPER | G protein-coupled estrogen receptor 1 | 2.509 | 3.11E-03 | Plasma Membrane | G-protein coupled receptor |
| SLC22A18 | solute carrier family 22, member 18 | 2.498 | 5.55E-03 | Plasma Membrane | transporter |
| FBLN7 | fibulin 7 | 2.498 | 4.13E-03 | Extracellular Space | other |
| PLTP | phospholipid transfer protein | 2.486 | 1.47E-03 | Extracellular Space | enzyme |
| TMEM47 | transmembrane protein 47 | 2.477 | 1.45E-02 | Plasma Membrane | other |
| OAF | OAF homolog (Drosophila) | 2.474 | 1.06E-03 | Other | other |
| PKDCC | protein kinase domain containing, cytoplasmic | 2.472 | 6.40E-03 | Cytoplasm | kinase |
| TMEM158 | transmembrane protein 158 (gene/pseudogene) | 2.466 | 4.74E-03 | Plasma Membrane | other |
| CREG1 | cellular repressor of E1A-stimulated genes 1 | 2.455 | 2.56E-03 | Nucleus | transcription regulator |
| PRKCDBP | protein kinase C, delta binding protein | 2.439 | 2.56E-03 | Cytoplasm | other |
| MST1 | macrophage stimulating 1 (hepatocyte growth factor-like) | 2.437 | 4.04E-03 | Extracellular Space | growth factor |
| DENND2A | DENN/MADD domain containing 2A | 2.425 | 6.67E-03 | Other | other |
| SPOCK1 | sparc/osteonectin, cwcv and kazal-like domains proteoglycan (testican) 1 | 2.424 | 1.92E-02 | Extracellular Space | other |
| TNFRSF25 | tumor necrosis factor receptor superfamily, member 25 | 2.421 | 1.07E-02 | Plasma Membrane | transmembrane receptor |
| FAM110B | family with sequence similarity 110, member B | 2.42 | 5.98E-03 | Cytoplasm | other |
| SLC15A3 | solute carrier family 15, member 3 | 2.418 | 3.80E-02 | Cytoplasm | transporter |
| CAPN5 | calpain 5 | 2.417 | 1.47E-03 | Cytoplasm | peptidase |
| CHI3L2 | chitinase 3-like 2 | 2.406 | 1.09E-03 | Extracellular Space | enzyme |
| FZD8 | frizzled family receptor 8 | 2.384 | 1.17E-02 | Plasma Membrane | G-protein coupled receptor |
| SMIM3 | small integral membrane protein 3 | 2.384 | 2.64E-03 | Other | ion channel |
| VPS9D1 | VPS9 domain containing 1 | 2.383 | 2.51E-03 | Other | transporter |
| TMEM132B | transmembrane protein 132B | 2.375 | 3.08E-02 | Other | other |
| CCDC151 | coiled-coil domain containing 151 | 2.359 | 5.03E-03 | Other | other |
| NDRG1 | N-myc downstream regulated 1 | 2.357 | 2.70E-03 | Nucleus | kinase |
| IDUA | iduronidase, alpha-L- | 2.356 | 6.56E-04 | Cytoplasm | enzyme |
| LOC339192 | uncharacterized LOC339192 | 2.356 | 3.48E-03 | Other | other |
| KIAA1644 | KIAA1644 | 2.348 | 2.16E-03 | Other | other |
| PDE7B | phosphodiesterase 7B | 2.333 | 1.96E-02 | Cytoplasm | enzyme |
| CA12 | carbonic anhydrase XII | 2.329 | 2.92E-02 | Plasma Membrane | enzyme |
| BHLHE40 | basic helix-loop-helix family, member e40 | 2.328 | 6.84E-03 | Nucleus | transcription regulator |
| SOBP | sine oculis binding protein homolog (Drosophila) | 2.314 | 1.79E-03 | Nucleus | other |
| MOB3C | MOB kinase activator 3C | 2.306 | 5.74E-03 | Other | other |
| CECR1 | cat eye syndrome chromosome region, candidate 1 | 2.304 | 1.73E-02 | Extracellular Space | enzyme |
| PFKFB4 | 6-phosphofructo-2-kinase/fructose-2,6-biphosphatase 4 | 2.295 | 1.50E-02 | Cytoplasm | kinase |
| MICAL1 | microtubule associated monooxygenase, calponin and LIM domain containing 1 | 2.29 | 3.90E-03 | Cytoplasm | enzyme |
| PLA2G4C | phospholipase A2, group IVC (cytosolic, calcium-independent) | 2.289 | 1.89E-03 | Plasma Membrane | enzyme |
| ZSWIM5 | zinc finger, SWIM-type containing 5 | 2.281 | 2.79E-03 | Other | other |
| CRIPAK | cysteine-rich PAK1 inhibitor | 2.272 | 1.78E-03 | Other | other |
| TMEM132A | transmembrane protein 132A | 2.268 | 3.82E-02 | Cytoplasm | other |
| JAM2 | junctional adhesion molecule 2 | 2.268 | 8.88E-04 | Plasma Membrane | other |
| TREM1 | triggering receptor expressed on myeloid cells 1 | 2.267 | 4.06E-03 | Plasma Membrane | transmembrane receptor |
| TMEM200A | transmembrane protein 200A | 2.265 | 3.17E-02 | Other | other |
| FNDC1 | fibronectin type III domain containing 1 | 2.262 | 8.88E-04 | Plasma Membrane | other |
| LARGE | like-glycosyltransferase | 2.258 | 1.89E-03 | Cytoplasm | enzyme |
| TMEM178A | transmembrane protein 178A | 2.248 | 1.49E-03 | Other | other |
| HGF | hepatocyte growth factor (hepapoietin A; scatter factor) | 2.246 | 6.81E-03 | Extracellular Space | growth factor |
| PPAP2A | phosphatidic acid phosphatase type 2A | 2.245 | 2.12E-02 | Plasma Membrane | phosphatase |
| BTG1 | B-cell translocation gene 1, anti-proliferative | 2.241 | 2.44E-03 | Nucleus | transcription regulator |
| MAPK13 | mitogen-activated protein kinase 13 | 2.239 | 8.57E-03 | Cytoplasm | kinase |
| TTYH2 | tweety homolog 2 (Drosophila) | 2.238 | 3.65E-03 | Other | ion channel |
| CYP26B1 | cytochrome P450, family 26, subfamily B, polypeptide 1 | 2.237 | 3.64E-03 | Cytoplasm | enzyme |
| HLA-B | major histocompatibility complex, class I, B | 2.236 | 9.66E-03 | Plasma Membrane | transmembrane receptor |
| ALPL | alkaline phosphatase, liver/bone/kidney | 2.235 | 4.41E-02 | Plasma Membrane | phosphatase |
| MIB2 | mindbomb E3 ubiquitin protein ligase 2 | 2.234 | 1.83E-03 | Nucleus | transcription regulator |
| GHDC | GH3 domain containing | 2.228 | 2.96E-03 | Cytoplasm | other |
| OLFML3 | olfactomedin-like 3 | 2.227 | 1.14E-02 | Extracellular Space | other |
| PANX2 | pannexin 2 | 2.225 | 3.30E-02 | Plasma Membrane | transporter |
| ABHD17C | abhydrolase domain containing 17C | 2.222 | 8.36E-03 | Other | enzyme |
| JUNB | jun B proto-oncogene | 2.219 | 2.26E-03 | Nucleus | transcription regulator |
| STARD8 | StAR-related lipid transfer (START) domain containing 8 | 2.212 | 2.36E-03 | Cytoplasm | other |
| PDLIM3 | PDZ and LIM domain 3 | 2.211 | 2.43E-02 | Cytoplasm | other |
| GSTM3 | glutathione S-transferase mu 3 (brain) | 2.199 | 2.74E-03 | Cytoplasm | enzyme |
| IFI44L | interferon-induced protein 44-like | 2.189 | 1.94E-02 | Other | other |
| SHC4 | SHC (Src homology 2 domain containing) family, member 4 | 2.183 | 8.73E-03 | Cytoplasm | other |
| LRFN5 | leucine rich repeat and fibronectin type III domain containing 5 | 2.182 | 2.70E-03 | Nucleus | other |
| FCGRT | Fc fragment of IgG, receptor, transporter, alpha | 2.173 | 8.88E-04 | Plasma Membrane | transmembrane receptor |
| CDC42EP5 | CDC42 effector protein (Rho GTPase binding) 5 | 2.171 | 1.37E-02 | Cytoplasm | other |
| GEM | GTP binding protein overexpressed in skeletal muscle | 2.166 | 6.98E-03 | Plasma Membrane | enzyme |
| A4GALT | alpha 1,4-galactosyltransferase | 2.164 | 1.41E-02 | Cytoplasm | enzyme |
| TNFRSF14 | tumor necrosis factor receptor superfamily, member 14 | 2.162 | 4.47E-02 | Plasma Membrane | transmembrane receptor |
| CD248 | CD248 molecule, endosialin | 2.159 | 1.86E-02 | Plasma Membrane | other |
| BRSK1 | BR serine/threonine kinase 1 | 2.155 | 3.95E-03 | Cytoplasm | kinase |
| IRF1 | interferon regulatory factor 1 | 2.146 | 2.21E-02 | Nucleus | transcription regulator |
| ANKRD37 | ankyrin repeat domain 37 | 2.144 | 2.48E-02 | Other | other |
| NUPR1 | nuclear protein, transcriptional regulator, 1 | 2.133 | 6.96E-03 | Nucleus | transcription regulator |
| CLEC11A | C-type lectin domain family 11, member A | 2.132 | 7.74E-03 | Extracellular Space | growth factor |
| PLIN2 | perilipin 2 | 2.118 | 3.95E-03 | Plasma Membrane | other |
| PDK4 | pyruvate dehydrogenase kinase, isozyme 4 | 2.117 | 3.99E-02 | Cytoplasm | kinase |
| PTPRM | protein tyrosine phosphatase, receptor type, M | 2.115 | 2.56E-03 | Plasma Membrane | phosphatase |
| HLA-H | major histocompatibility complex, class I, H (pseudogene) | 2.114 | 3.86E-02 | Other | other |
| SLC1A1 | solute carrier family 1 (neuronal/epithelial high affinity glutamate transporter, system Xag), member 1 | 2.108 | 5.90E-03 | Plasma Membrane | transporter |
| EFNB3 | ephrin-B3 | 2.105 | 4.71E-03 | Plasma Membrane | kinase |
| UBA7 | ubiquitin-like modifier activating enzyme 7 | 2.103 | 2.60E-02 | Cytoplasm | enzyme |
| SIX5 | SIX homeobox 5 | 2.102 | 3.52E-03 | Nucleus | transcription regulator |
| GABRE | gamma-aminobutyric acid (GABA) A receptor, epsilon | 2.098 | 1.89E-03 | Plasma Membrane | ion channel |
| NES | nestin | 2.091 | 1.71E-02 | Cytoplasm | other |
| MILR1 | mast cell immunoglobulin-like receptor 1 | 2.088 | 2.16E-02 | Other | other |
| HBEGF | heparin-binding EGF-like growth factor | 2.084 | 1.96E-02 | Extracellular Space | growth factor |
| RHBDF1 | rhomboid 5 homolog 1 (Drosophila) | 2.079 | 1.96E-03 | Other | other |
| HLA-F | major histocompatibility complex, class I, F | 2.078 | 6.27E-03 | Plasma Membrane | transmembrane receptor |
| ARHGEF19 | Rho guanine nucleotide exchange factor (GEF) 19 | 2.078 | 3.06E-03 | Other | other |
| DCN | decorin | 2.063 | 4.16E-02 | Extracellular Space | other |
| DDR1 | discoidin domain receptor tyrosine kinase 1 | 2.062 | 1.15E-02 | Plasma Membrane | kinase |
| SLC27A3 | solute carrier family 27 (fatty acid transporter), member 3 | 2.055 | 3.67E-03 | Cytoplasm | transporter |
| LTBP3 | latent transforming growth factor beta binding protein 3 | 2.055 | 1.58E-03 | Extracellular Space | other |
| COL18A1 | collagen, type XVIII, alpha 1 | 2.054 | 3.30E-02 | Extracellular Space | other |
| NQO2 | NAD(P)H dehydrogenase, quinone 2 | 2.053 | 2.94E-03 | Cytoplasm | enzyme |
| IL7R | interleukin 7 receptor | 2.046 | 3.20E-02 | Plasma Membrane | transmembrane receptor |
| ATP6V1B1 | ATPase, H+ transporting, lysosomal 56/58kDa, V1 subunit B1 | 2.044 | 1.79E-03 | Cytoplasm | transporter |
| VLDLR | very low density lipoprotein receptor | 2.042 | 9.56E-03 | Plasma Membrane | transporter |
| SLC6A10P | solute carrier family 6 (neurotransmitter transporter, creatine), member 10, pseudogene | 2.039 | 2.51E-03 | Plasma Membrane | transporter |
| DDIT3 | DNA-damage-inducible transcript 3 | 2.038 | 5.61E-03 | Nucleus | transcription regulator |
| GPR124 | G protein-coupled receptor 124 | 2.036 | 1.97E-02 | Plasma Membrane | G-protein coupled receptor |
| RNASE4 | ribonuclease, RNase A family, 4 | 2.031 | 7.48E-03 | Extracellular Space | enzyme |
| MAPK8IP3 | mitogen-activated protein kinase 8 interacting protein 3 | 2.029 | 3.56E-02 | Cytoplasm | other |
| WIPI1 | WD repeat domain, phosphoinositide interacting 1 | 2.027 | 3.46E-02 | Cytoplasm | other |
| MZF1 | myeloid zinc finger 1 | 2.026 | 3.40E-03 | Nucleus | transcription regulator |
| IER5L | immediate early response 5-like | 2.024 | 7.07E-03 | Other | other |
| QPCT | glutaminyl-peptide cyclotransferase | 2.023 | 1.62E-02 | Cytoplasm | enzyme |
| LOXL3 | lysyl oxidase-like 3 | 2.021 | 1.62E-02 | Extracellular Space | enzyme |
| TRIM47 | tripartite motif containing 47 | 2.02 | 3.79E-03 | Cytoplasm | other |
| NPEPL1 | aminopeptidase-like 1 | 2.018 | 5.42E-03 | Other | peptidase |
| SLIT3 | slit homolog 3 (Drosophila) | 2.016 | 1.45E-02 | Extracellular Space | other |
| NMB | neuromedin B | 2.015 | 5.61E-03 | Extracellular Space | other |
| PDGFD | platelet derived growth factor D | 2.011 | 1.85E-02 | Extracellular Space | growth factor |
| SNTB1 | syntrophin, beta 1 (dystrophin-associated protein A1, 59kDa, basic component 1) | 2.01 | 5.61E-03 | Plasma Membrane | other |
| RCN3 | reticulocalbin 3, EF-hand calcium binding domain | 2.006 | 1.97E-02 | Cytoplasm | other |
| CHI3L1 | chitinase 3-like 1 (cartilage glycoprotein-39) | 2.003 | 8.88E-04 | Extracellular Space | enzyme |
| EMILIN2 | elastin microfibril interfacer 2 | 2 | 1.28E-02 | Extracellular Space | other |

**Supplementary Table 5c**

Genes up in ckit k-med vs CSps

| **Symbol** | **Entrez Gene Name** | **Log Ratio** | **p-value** | **Location** | **Type(s)** |
| --- | --- | --- | --- | --- | --- |
| NPPB | natriuretic peptide B | 5.507 | 1.63E-02 | Extracellular Space | other |
| CLDN1 | claudin 1 | 5.02 | 4.77E-02 | Plasma Membrane | other |
| ALPK2 | alpha-kinase 2 | 4.968 | 1.52E-02 | Other | kinase |
| ANKRD1 | ankyrin repeat domain 1 (cardiac muscle) | 3.963 | 2.33E-02 | Cytoplasm | transcription regulator |
| PSAT1 | phosphoserine aminotransferase 1 | 3.953 | 1.52E-02 | Cytoplasm | enzyme |
| CPA4 | carboxypeptidase A4 | 3.939 | 3.04E-02 | Extracellular Space | peptidase |
| KRT7 | keratin 7 | 3.907 | 4.55E-02 | Cytoplasm | other |
| TAGLN | transgelin | 3.576 | 1.52E-02 | Cytoplasm | other |
| FOXD1 | forkhead box D1 | 3.225 | 4.06E-02 | Nucleus | transcription regulator |
| STC2 | stanniocalcin 2 | 3.168 | 2.19E-02 | Extracellular Space | other |
| CHAC1 | ChaC, cation transport regulator homolog 1 (E. coli) | 3.149 | 1.52E-02 | Cytoplasm | other |
| RAB3B | RAB3B, member RAS oncogene family | 3.111 | 1.12E-02 | Cytoplasm | enzyme |
| TUFT1 | tuftelin 1 | 2.991 | 4.26E-02 | Other | other |
| ASNS | asparagine synthetase (glutamine-hydrolyzing) | 2.803 | 1.29E-02 | Cytoplasm | enzyme |
| THBS1 | thrombospondin 1 | 2.619 | 1.52E-02 | Extracellular Space | other |
| ENC1 | ectodermal-neural cortex 1 (with BTB domain) | 2.615 | 2.19E-02 | Nucleus | peptidase |
| DDAH1 | dimethylarginine dimethylaminohydrolase 1 | 2.543 | 1.63E-02 | Cytoplasm | enzyme |
| RGS4 | regulator of G-protein signaling 4 | 2.527 | 2.55E-02 | Cytoplasm | other |
| PPP1R3C | protein phosphatase 1, regulatory subunit 3C | 2.52 | 1.87E-02 | Cytoplasm | phosphatase |
| SLC25A4 | solute carrier family 25 (mitochondrial carrier; adenine nucleotide translocator), member 4 | 2.321 | 4.59E-02 | Cytoplasm | transporter |
| CALD1 | caldesmon 1 | 2.295 | 3.17E-02 | Cytoplasm | other |
| CDC42EP3 | CDC42 effector protein (Rho GTPase binding) 3 | 2.281 | 1.12E-02 | Cytoplasm | other |
| NEXN | nexilin (F actin binding protein) | 2.275 | 1.63E-02 | Plasma Membrane | other |
| PAWR | PRKC, apoptosis, WT1, regulator | 2.267 | 4.27E-02 | Nucleus | transcription regulator |
| CRIM1 | cysteine rich transmembrane BMP regulator 1 (chordin-like) | 2.247 | 4.03E-02 | Extracellular Space | kinase |
| UBASH3B | ubiquitin associated and SH3 domain containing B | 2.204 | 1.92E-02 | Other | enzyme |
| FRMD6 | FERM domain containing 6 | 2.164 | 1.12E-02 | Cytoplasm | other |
| AMY1A (includes others) | amylase, alpha 1A (salivary) | 2.119 | 3.45E-02 | Extracellular Space | enzyme |
| NUAK1 | NUAK family, SNF1-like kinase, 1 | 2.1 | 4.03E-02 | Other | kinase |
| AHNAK2 | AHNAK nucleoprotein 2 | 2.1 | 4.22E-02 | Other | other |
| AJUBA | ajuba LIM protein | 2.049 | 2.61E-02 | Nucleus | transcription regulator |
| EEF1A2 | eukaryotic translation elongation factor 1 alpha 2 | 2.042 | 4.21E-02 | Cytoplasm | translation regulator |

Genes up in CSps vs kit k-med

| **Symbol** | **Entrez Gene Name** | **Log Ratio** | **p-value** | **Location** | **Type(s)** |
| --- | --- | --- | --- | --- | --- |
| RARRES2 | retinoic acid receptor responder (tazarotene induced) 2 | 6.851 | 1.88E-03 | Plasma Membrane | transmembrane receptor |
| APOE | apolipoprotein E | 5.99 | 4.03E-03 | Extracellular Space | transporter |
| GPNMB | glycoprotein (transmembrane) nmb | 5.405 | 4.03E-03 | Plasma Membrane | enzyme |
| MGP | matrix Gla protein | 5.316 | 3.79E-02 | Extracellular Space | other |
| TGFB3 | transforming growth factor, beta 3 | 5.099 | 1.88E-03 | Extracellular Space | growth factor |
| IL24 | interleukin 24 | 4.972 | 2.09E-02 | Extracellular Space | cytokine |
| CST1 | cystatin SN | 4.899 | 3.61E-02 | Other | other |
| COL7A1 | collagen, type VII, alpha 1 | 4.357 | 2.82E-02 | Extracellular Space | other |
| RASD1 | RAS, dexamethasone-induced 1 | 4.309 | 3.76E-02 | Cytoplasm | enzyme |
| CRLF1 | cytokine receptor-like factor 1 | 4.28 | 1.12E-02 | Extracellular Space | other |
| DCN | decorin | 4.149 | 6.43E-03 | Extracellular Space | other |
| PTGDS | prostaglandin D2 synthase 21kDa (brain) | 4.052 | 4.03E-03 | Cytoplasm | enzyme |
| TACSTD2 | tumor-associated calcium signal transducer 2 | 3.879 | 1.88E-03 | Plasma Membrane | other |
| FOS | FBJ murine osteosarcoma viral oncogene homolog | 3.844 | 2.00E-02 | Nucleus | transcription regulator |
| NKD2 | naked cuticle homolog 2 (Drosophila) | 3.818 | 2.71E-02 | Nucleus | other |
| NR4A2 | nuclear receptor subfamily 4, group A, member 2 | 3.733 | 1.88E-03 | Nucleus | ligand-dependent nuclear receptor |
| FBLN1 | fibulin 1 | 3.648 | 2.44E-02 | Extracellular Space | other |
| GPX3 | glutathione peroxidase 3 (plasma) | 3.583 | 3.52E-02 | Extracellular Space | enzyme |
| VWCE | von Willebrand factor C and EGF domains | 3.581 | 2.60E-03 | Other | other |
| PAPLN | papilin, proteoglycan-like sulfated glycoprotein | 3.546 | 1.88E-03 | Extracellular Space | other |
| NDP | Norrie disease (pseudoglioma) | 3.526 | 2.19E-02 | Extracellular Space | growth factor |
| CALCRL | calcitonin receptor-like | 3.478 | 1.88E-03 | Plasma Membrane | G-protein coupled receptor |
| FGL2 | fibrinogen-like 2 | 3.432 | 2.55E-02 | Extracellular Space | peptidase |
| IFITM1 | interferon induced transmembrane protein 1 | 3.412 | 1.63E-02 | Plasma Membrane | transmembrane receptor |
| C2CD4B | C2 calcium-dependent domain containing 4B | 3.22 | 1.12E-02 | Other | other |
| PARPBP | PARP1 binding protein | 3.161 | 3.17E-02 | Nucleus | other |
| NPTX2 | neuronal pentraxin II | 3.057 | 4.27E-02 | Extracellular Space | other |
| EDNRB | endothelin receptor type B | 2.946 | 1.28E-02 | Plasma Membrane | G-protein coupled receptor |
| COLEC12 | collectin sub-family member 12 | 2.859 | 2.40E-02 | Plasma Membrane | transmembrane receptor |
| OAS2 | 2'-5'-oligoadenylate synthetase 2, 69/71kDa | 2.858 | 2.00E-02 | Cytoplasm | enzyme |
| CPXM2 | carboxypeptidase X (M14 family), member 2 | 2.806 | 4.06E-02 | Extracellular Space | peptidase |
| HEY1 | hairy/enhancer-of-split related with YRPW motif 1 | 2.799 | 1.63E-02 | Nucleus | transcription regulator |
| PLTP | phospholipid transfer protein | 2.792 | 1.61E-02 | Extracellular Space | enzyme |
| ISG20 | interferon stimulated exonuclease gene 20kDa | 2.772 | 3.52E-02 | Nucleus | enzyme |
| EMILIN1 | elastin microfibril interfacer 1 | 2.735 | 6.43E-03 | Extracellular Space | other |
| SLC22A17 | solute carrier family 22, member 17 | 2.729 | 2.09E-02 | Plasma Membrane | transporter |
| IGFBP1 | insulin-like growth factor binding protein 1 | 2.666 | 1.78E-02 | Extracellular Space | other |
| C1QTNF6 | C1q and tumor necrosis factor related protein 6 | 2.621 | 4.70E-02 | Extracellular Space | other |
| SERPINF1 | serpin peptidase inhibitor, clade F (alpha-2 antiplasmin, pigment epithelium derived factor), member 1 | 2.6 | 1.63E-02 | Extracellular Space | other |
| ADCY4 | adenylate cyclase 4 | 2.573 | 6.19E-03 | Plasma Membrane | enzyme |
| IFI44L | interferon-induced protein 44-like | 2.487 | 1.63E-02 | Other | other |
| CCDC151 | coiled-coil domain containing 151 | 2.481 | 3.74E-02 | Other | other |
| C1R | complement component 1, r subcomponent | 2.39 | 2.00E-02 | Extracellular Space | peptidase |
| IL13RA2 | interleukin 13 receptor, alpha 2 | 2.382 | 6.92E-03 | Plasma Membrane | transmembrane receptor |
| MAPK13 | mitogen-activated protein kinase 13 | 2.379 | 4.07E-02 | Cytoplasm | kinase |
| LRFN5 | leucine rich repeat and fibronectin type III domain containing 5 | 2.362 | 2.00E-02 | Nucleus | other |
| RFTN2 | raftlin family member 2 | 2.354 | 2.00E-02 | Other | other |
| TTYH2 | tweety homolog 2 (Drosophila) | 2.328 | 3.63E-02 | Other | ion channel |
| SOBP | sine oculis binding protein homolog (Drosophila) | 2.309 | 1.52E-02 | Nucleus | other |
| LDB2 | LIM domain binding 2 | 2.307 | 4.03E-03 | Nucleus | transcription regulator |
| RASIP1 | Ras interacting protein 1 | 2.286 | 4.23E-02 | Cytoplasm | other |
| PKDCC | protein kinase domain containing, cytoplasmic | 2.282 | 3.04E-02 | Cytoplasm | kinase |
| PRKCDBP | protein kinase C, delta binding protein | 2.251 | 4.35E-02 | Cytoplasm | other |
| TRIM47 | tripartite motif containing 47 | 2.237 | 1.89E-02 | Cytoplasm | other |
| CD93 | CD93 molecule | 2.221 | 2.55E-02 | Plasma Membrane | other |
| ADAMTS9 | ADAM metallopeptidase with thrombospondin type 1 motif, 9 | 2.217 | 3.17E-02 | Extracellular Space | peptidase |
| CHI3L2 | chitinase 3-like 2 | 2.211 | 2.00E-02 | Extracellular Space | enzyme |
| EFNB3 | ephrin-B3 | 2.21 | 4.03E-02 | Plasma Membrane | kinase |
| STARD8 | StAR-related lipid transfer (START) domain containing 8 | 2.178 | 6.43E-03 | Cytoplasm | other |
| TWIST1 | twist basic helix-loop-helix transcription factor 1 | 2.149 | 3.76E-02 | Nucleus | transcription regulator |
| SOD2 | superoxide dismutase 2, mitochondrial | 2.129 | 3.48E-02 | Cytoplasm | enzyme |
| NR1H3 | nuclear receptor subfamily 1, group H, member 3 | 2.118 | 2.00E-02 | Nucleus | ligand-dependent nuclear receptor |
| ATP6V1B1 | ATPase, H+ transporting, lysosomal 56/58kDa, V1 subunit B1 | 2.079 | 1.63E-02 | Cytoplasm | transporter |
| TMEM59L | transmembrane protein 59-like | 2.068 | 4.98E-02 | Cytoplasm | other |
| CTSL | cathepsin L | 2.06 | 1.63E-02 | Cytoplasm | peptidase |
| PDXDC2P | pyridoxal-dependent decarboxylase domain containing 2, pseudogene | 2.052 | 1.63E-02 | Other | other |
| SOX17 | SRY (sex determining region Y)-box 17 | 2.044 | 5.04E-02 | Nucleus | transcription regulator |
| SLC27A3 | solute carrier family 27 (fatty acid transporter), member 3 | 2.022 | 2.55E-02 | Cytoplasm | transporter |
| NRCAM | neuronal cell adhesion molecule | 2.017 | 3.61E-02 | Plasma Membrane | other |
| IFI6 | interferon, alpha-inducible protein 6 | 2.01 | 2.61E-02 | Cytoplasm | other |
| RRAGD | Ras-related GTP binding D | 2.01 | 1.63E-02 | Cytoplasm | enzyme |
| KIAA1211 | KIAA1211 | 2.006 | 1.52E-02 | Other | other |
