# Supplementary material for: Different types of cultured human adult Cardiac Progenitor Cells have a high degree of transcriptome similarity
Source: J Cell Mol Med. 2014 Oct 14;18(11):2147–51. doi: 10.1111/jcmm.12458 (PMC4224548; doi:10.1111/jcmm.12458)
Supplement: Table S6 — Ingenuity molecular networks analysis gene list. [file jcmm0018-2147-sd8.doc]

**Supplementary Table 6a: Differentially regulated genes between Sca GEL SP++ and CSps in Cardiovascular System Development and Function, Embryonic Development, Organismal Development Nethwork.**

| **Entrez Gene Name** | **Entrez Gene** | **Log Ratio** | **p-value** | **Location** | **Family** |
| --- | --- | --- | --- | --- | --- |
| bone morphogenetic protein 2 | BMP2 | -3.0604 | 0.0471 | Extracellular Space | growth factor |
| chemokine (C-C motif) ligand 7 | CCL7 | -5.2421 | 0.0001 | Extracellular Space | cytokine |
| CDC42 effector protein (Rho GTPase binding) 5 | CDC42EP5 | -2.1710 | 0.0137 | Cytoplasm | other |
| complement factor B | CFB | -3.0754 | 0.0128 | Extracellular Space | peptidase |
| collagen, type XVIII, alpha 1 | COL18A1 | -2.0543 | 0.0330 | Extracellular Space | other |
| ephrin-B2 | EFNB2 | 3.2753 | 0.0177 | Plasma Membrane | other |
| early growth response 2 | EGR2 | -6.0263 | 0.0001 | Nucleus | transcription regulator |
| GTP binding protein overexpressed in skeletal muscle | GEM | -2.1659 | 0.0070 | Plasma Membrane | enzyme |
| G protein-coupled estrogen receptor 1 | GPER | -2.5088 | 0.0031 | Plasma Membrane | G-protein coupled receptor |
| heparin-binding EGF-like growth factor | HBEGF | -2.0841 | 0.0196 | Extracellular Space | growth factor |
| hepatocyte growth factor (hepapoietin A; scatter factor) | HGF | -2.2461 | 0.0068 | Extracellular Space | growth factor |
| jun B proto-oncogene | JUNB | -2.2190 | 0.0023 | Nucleus | transcription regulator |
| leukemia inhibitory factor | LIF | -3.9758 | 0.0023 | Extracellular Space | cytokine |
| matrix Gla protein | MGP | -5.0330 | 0.0021 | Extracellular Space | other |
| matrix metallopeptidase 10 (stromelysin 2) | MMP10 | -3.4751 | 0.0041 | Extracellular Space | peptidase |
| matrix metallopeptidase 2 (gelatinase A, 72kDa gelatinase, 72kDa type IV collagenase) | MMP2 | -2.8375 | 0.0022 | Extracellular Space | peptidase |
| N-myc downstream regulated 1 | NDRG1 | -2.3566 | 0.0027 | Nucleus | kinase |
| nexilin (F actin binding protein) | NEXN | 2.8122 | 0.0012 | Plasma Membrane | other |
| nuclear receptor subfamily 4, group A, member 2 | NR4A2 | -4.3011 | 0.0002 | Nucleus | ligand-dependent nuclear receptor |
| platelet-derived growth factor receptor, beta polypeptide | PDGFRB | -3.0827 | 0.0086 | Plasma Membrane | kinase |
| pyruvate dehydrogenase kinase, isozyme 4 | PDK4 | -2.1172 | 0.0399 | Cytoplasm | kinase |
| 6-phosphofructo-2-kinase/fructose-2,6-biphosphatase 4 | PFKFB4 | -2.2951 | 0.0150 | Cytoplasm | kinase |
| runt-related transcription factor 2 | RUNX2 | 2.3867 | 0.0060 | Nucleus | transcription regulator |
| serpin peptidase inhibitor, clade B (ovalbumin), member 2 | SERPINB2 | 2.8221 | 0.0203 | Extracellular Space | other |
| thrombospondin 1 | THBS1 | 2.2612 | 0.0063 | Extracellular Space | other |
| transmembrane protein 158 (gene/pseudogene) | TMEM158 | -2.4661 | 0.0047 | Plasma Membrane | other |
| triggering receptor expressed on myeloid cells 1 | TREM1 | -2.2669 | 0.0041 | Plasma Membrane | transmembrane receptor |
| vascular endothelial growth factor A | VEGFA | -3.5470 | 0.0007 | Extracellular Space | growth factor |

**Supplementary Table 6b:** Differentially regulated genes between CSps vs CDCs FN CEM in Cardiovascular System Development and Function, Organismal Development, Cell-To-Cell Signaling and Interaction network.

| **Entrez Gene Name** | **Gene symbol** | **Log Ratio** | **p-value** | **Location** | **Family** |
| --- | --- | --- | --- | --- | --- |
| apolipoprotein E | APOE | -5.6499 | 0.0028 | Extracellular Space | transporter |
| bradykinin receptor B2 | BDKRB2 | -2.2147 | 0.0494 | Plasma Membrane | G-protein coupled receptor |
| bone morphogenetic protein 2 | BMP2 | -2.5618 | 0.0083 | Extracellular Space | growth factor |
| collagen, type XVIII, alpha 1 | COL18A1 | -2.3315 | 0.0035 | Extracellular Space | other |
| early growth response 2 | EGR2 | -4.7939 | 0.0039 | Nucleus | transcription regulator |
| gamma-aminobutyric acid (GABA) B receptor, 2 | GABBR2 | -2.7330 | 0.0451 | Plasma Membrane | G-protein coupled receptor |
| hepatocyte growth factor (hepapoietin A; scatter factor) | HGF | -2.4865 | 0.0222 | Extracellular Space | growth factor |
| interleukin 11 | IL11 | -4.9912 | 0.0002 | Extracellular Space | cytokine |
| interleukin 33 | IL33 | -2.6326 | 0.0125 | Extracellular Space | cytokine |
| leukemia inhibitory factor | LIF | -2.3190 | 0.0223 | Extracellular Space | cytokine |
| matrix Gla protein | MGP | -5.9090 | 0.0045 | Extracellular Space | other |
| platelet-derived growth factor receptor, beta polypeptide | PDGFRB | -3.0311 | 0.0047 | Plasma Membrane | kinase |
| pyruvate dehydrogenase kinase, isozyme 4 | PDK4 | -2.9296 | 0.0041 | Cytoplasm | kinase |
| plasminogen activator, tissue | PLAT | -2.2283 | 0.0125 | Extracellular Space | peptidase |
| prostaglandin E receptor 2 (subtype EP2), 53kDa | PTGER2 | -2.2769 | 0.0285 | Plasma Membrane | G-protein coupled receptor |
| prostaglandin E synthase | PTGES | -3.3140 | 0.0145 | Cytoplasm | enzyme |
| prostaglandin-endoperoxide synthase 2 (prostaglandin G/H synthase and cyclooxygenase) | PTGS2 | -2.7471 | 0.0039 | Cytoplasm | enzyme |
| transforming growth factor, beta 2 | TGFB2 | 2.1231 | 0.0345 | Extracellular Space | growth factor |
| thrombospondin 1 | THBS1 | 2.1166 | 0.0233 | Extracellular Space | other |

**Supplementary Table 6c:** Differentially regulated genes between CSps and Kit K-Med in Cardiovascular System Development and Function, Organismal Development, Tissue Morphology network.

| **Entrez Gene Name** | **Gene symbol** | **Log Ratio** | **p-value** | **Location** | **Family** |
| --- | --- | --- | --- | --- | --- |
| apolipoprotein E | APOE | -5.9900 | 0.0040 | Extracellular Space | transporter |
| caldesmon 1 | CALD1 | 2.2949 | 0.0317 | Cytoplasm | other |
| forkhead box D1 | FOXD1 | 3.2248 | 0.0406 | Nucleus | transcription regulator |
| mitogen-activated protein kinase 13 | MAPK13 | -2.3785 | 0.0407 | Cytoplasm | kinase |
| matrix Gla protein | MGP | -5.3156 | 0.0379 | Extracellular Space | other |
| naked cuticle homolog 2 (Drosophila) | NKD2 | -3.8181 | 0.0271 | Nucleus | other |
| nuclear receptor subfamily 4, group A, member 2 | NR4A2 | -3.7326 | 0.0019 | Nucleus | ligand-dependent nuclear receptor |
| phospholipid transfer protein | PLTP | -2.7919 | 0.0161 | Extracellular Space | enzyme |
| protein phosphatase 1, regulatory subunit 3C | PPP1R3C | 2.5196 | 0.0187 | Cytoplasm | phosphatase |
| phosphoserine aminotransferase 1 | PSAT1 | 3.9530 | 0.0152 | Cytoplasm | enzyme |
| serpin peptidase inhibitor, clade F (alpha-2 antiplasmin, pigment epithelium derived factor), member 1 | SERPINF1 | -2.6003 | 0.0163 | Extracellular Space | other |
