# Supplementary material for: Different types of cultured human adult Cardiac Progenitor Cells have a high degree of transcriptome similarity
Source: J Cell Mol Med. 2014 Oct 14;18(11):2147–51. doi: 10.1111/jcmm.12458 (PMC4224548; doi:10.1111/jcmm.12458)
Supplement: Table S7 — Culture conditions of Isolated CPCs. [file jcmm0018-2147-sd9.docx]

| **Culture**  **condition**  **Cell type** | **Coating** | **Medium** |
| --- | --- | --- |
| Kit-CDCs GEL SP++  Sca-CDCs GEL SP++  Sca GEL SP++  CDCs GEL SP++  Kit GEL SP++ | Gelatin | - 25% EGM-2 (3% EGM-2 single quotes in EBM-2);  - 75% M199;  - 10% FBS;  - 1x MEM non-essential amino acids;  - 1x penicillin/streptomycin; |
| Kit-CDCs FN CEM  Sca-CDCs FN CEM  CDCs FN CEM | Fibronectin | - IMDM;  - 10% FBS;  - 1X penicillin/streptomycin;  - 1% l-glutamine;  - 0.1 mM 2-mercaptoethanol; |
| Kit K-Med | None | - HAM’S F12;  - 10% FBS;  - 0,2mM L-Glutatione;  - 5mU/ml human Erythropoietin;  - 10ng/ml basic FgF;  - 1x penicillin/streptomycin; |
| CSps | Poly-D-Lysine | - 35% IMDM;  - DMEM/F-12 Mix;  - 3.5% FBS;  - 2% B-27 supplement;  - 1 unit/mL Thrombin;  - 80 ng/mL bFGF;  - 25 ng/mL EGF;  - 4 ng/mL cardiotrophin-1;  - 1X penicillin/streptomycin;  - 1% l-glutamine;  - 0.1 mM 2-mercaptoethanol; |

Supplementary Table 7 : Culture conditions of Isolated CPCs.
