# Supplementary material for: Different types of cultured human adult Cardiac Progenitor Cells have a high degree of transcriptome similarity
Source: J Cell Mol Med. 2014 Oct 14;18(11):2147–51. doi: 10.1111/jcmm.12458 (PMC4224548; doi:10.1111/jcmm.12458)
Supplement: Supplementary file 10 [file jcmm0018-2147-sd10.doc]

**Supplementary Materials and Methods.**

*Cell isolation and Culture:*

Human cardiac progenitor cells were isolated from anonymous adult human auricle samples from 20 different patients Standard informed consent procedures and prior approval of the ethics committee of the University Medical Center Utrecht for surgical waist material were obtained. For direct c-Kit and Sca-1 isolation, the cardiac left auricles were cut in small pieces and enzymatically digested in 1 mg/ml collagenase A (Roche 103578) for 2 hours at 37°C. The digested tissue was placed in a 40µm cell strainer to obtain single cell suspension and CPCs were isolated by Magnetic cell sorting as described before (1,2).

Sca-1+ cells were plated at 0.1% gelatin coated wells in growth medium consisting of 25% EGM-2( 3% EGM-2 single quotes (Cambrex, cat. no. CC-4176) in EBM-2 (Cambrex, cat. no. CC-3156)) and 75% M199 (BioWhittaker, cat. no. BE12- 119F), 10% FBS (Hyclone, cat. no. CH30160.30), 1x MEM non-essential amino acids (BioWhittaker, cat. no. BE13-114E) and 1x penicillin/streptomycin (Sigma, cat. no. P4458) (2).

c-Kit+ cells were cultured in HAM’S F12 (Fisher, SH3002601), 10% FBS (Fisher, cat. no. SH3040602), 0,2mM L-Glutatione (Sigma, cat. no. G6013), 5mU/ml human Erythropoietin (Sigma, cat. no. E5627), 10ng/ml basic FgF (peprotech, cat. no. 100-18B) and 1x penicillin/streptomycin (Sigma, cat. no. P4458) (1).

Alternatively, human auricle biopsy samples were cut in small pieces and cultured as explants in 4 mg/mL Fibronectin coated dishes in Complete Explant Medium (CEM) consisting of IMDM (Gibco, cat. no.21980), 20% FBS (Hyclone, cat. no. CH30160.30), and 1x penicillin/streptomycin (Sigma, cat. no. P4458). Cardiosphere were cultured in Cardiosphere-growth medium (CGM): 35% IMDM and 65% DMEM/F-12 Mix, 3.5% FBS, 1% penicillin–streptomycin, 1% l-glutamine, 0.1 mM 2-mercaptoethanol, 1 unit/mL Thrombin (Sigma cat. no. T4393), 2% B-27 supplement (Invitrogen cat no. 17504-044), 80 ng/mL bFGF (Peprotech cat. no. 100-18B), 25 ng/mL EGF (Peprotech cat. no. AF-100-15) and 4 ng/mL cardiotrophin-1 (Peprotech cat. no. 300-32) (3). CDCs were cultured in CEM and Fibronectin coated flasks (4).

c-Kit (Miltenyi cat no. 130-091-332) and Sca-1+ (Miltenyi cat no. 130-092-529) cells were subsequently isolated by MiniMACS Magnetic cell sorting (Miltenyi Biotech) and cultured as above.

*RNA isolation, quality control, and Microarray analysis*:

Upon culture expansion, RNA was isolated by NucleoSpin RNAII column (Macherey-Nagel, cat no. 740955.250). RNA isolation was performed at passage 7 to 10 after isolation. The Quality control, RNA labeling, hybridization and data extraction were performed at ServiceXS B.V. (Leiden, The Netherlands). RNA concentration and purity was measured using the NanoDrop ND-1000 Spectrophotometer. Only samples with a 260/280 ratio between 1.8 and 2.1 were used for further analysis. RNA integrity was measured using the Agilent 2100 Bioanalyzer. After visual inspection of the Bioanalyzer electropherograms, only samples with a RNA Integrity Number (RIN) ≥ 9 were accepted for amplification and labeling process.

Amplification and labeling of the RNA samples was performed according to the manufacturer’s specifications. For Illumina RNA profiling experiments, the Ambion® Illumina TotalPrep RNA Amplification Kit (Ambion, cat no. IL1791) was used. Labelled cRNA was generated according to the Illumina “whole-genome gene expression direct hybridization assay” protocol and hybridized on Illumina HumanHT12-v4 Beadchips. Fluorescence intensity data obtained from Illumina Beadstudio was processed using the “R” bioconductor with the “lumi” package using variance-stabilizing transformation within technical replicates and robust spline normalization to normalize between samples. Quality control was performed on all microarrays to exclude technical errors and only genes that passed the detection call in >1 sample were included in the analysis.

Statistical analysis:

To identify differentially expressed genes, pairwise comparisons between progenitor cells obtained using different culturing methods were performed using the moderated T-test employed in the “limma” package (Smyth, G. K. et al. *Statistical applications in genetics and molecular biology*; 2004). The False Discovery Rate was controlled using the method by Benjamini and Hochberg (Benjamini, Y. Hochberg, Controlling the false discovery rate: a practical and powerful approach to multiple testing, Journal of the Royal Statistical Society. Series B; 1995). Sample relationships were investigated by hierarchical agglomerative clustering using the unweighted pair group with arithmetic mean (UPGMA) algorithm, with sample correlation as distance measure. For cluster analysis within defined cellular processes, a similar method employing Euclidean distance was used.
