# Supplementary material for: Different types of cultured human adult Cardiac Progenitor Cells have a high degree of transcriptome similarity
Source: J Cell Mol Med. 2014 Oct 14;18(11):2147–51. doi: 10.1111/jcmm.12458 (PMC4224548; doi:10.1111/jcmm.12458)
Supplement: Supplementary file 11 [file jcmm0018-2147-sd11.doc]

**Supplementary Figure 1.**

Representative pictures of individual cultures are presented for each used condition. A) Sca-1+ cells isolated from human auricle biopsy and cultured in gelatin coated flasks and Sca-1 medium (Sca GEL S-MED) (2); B) Sca-1+ CPCs cultured in c-Kit culture conditions (Sca K-MED) (1); C) C-Kit+ cells isolated from human auricle biopsy and cultured in Kit-CPCs Medium (Kit K-MED) (1); D) C-Kit+ cells cultured in gelatin coated flasks and Sca- medium (Kit GEL S-MED) (2); E) Cardiospheres (CSps) isolated from human auricle biopsy explant culture (3); F) Cardiospheres derived cells (CDCs) in Fibronectin and Complete Explant Medium (FN CEM) (4); G) c-kit+ or Sca-1+ cells isolated from CDCs FN CEM and cultured in FN CEM (Kit-CDCs FN/CEM (G) or Sca-CDCs FN/CEM (H)) or in GEL S MED condition (Kit-CDCs GEL S MED (I) or Sca-CDCs GEL S-MED (L). Scale bar= 20µm.

**Supplementary Figure 2.** Genome guided analysis of CPCs samples; Heat map of genes involved in (a) stem cell related pathways (TGF-ß, WNT, NFκB, p53, JAK/STAT, Notch, Hedgehog); (b) cell cycle and proliferation (G1 Phase and G1/S Transition; S Phase and DNA Replication; G2 Phase and G2/M Transition; M Phase; Cell Cycle Checkpoint and Arrest and Cell Cycle Regulators); (c) Stem cell transcription factors; (d) Growth Factors, Cytokines and Chemokines.

**Supplementary Table1:** CPCs samples and donors used in the project.

**Supplementary Table2:** pair-waise comparison of CPCs averaged values.

**Supplementary Table 3a:** Significantly Differentially expressed genes between monolayer growing CPCs groups.

**Supplementary Table 3b:** Significantly differentially expressed genes between CSps and monolayer growing CPCs.

**Supplementary Table 4:** List of significantly differentially expressed genes between monolayer growing CPCs.

**Supplementary Table 5a:** List of significantly differentially expressed genes between CSps and Sca GEL SP++.

**Supplementary Table 5b:** List of significantly differentially expressed genes between CSps and CDCs FN CEM.

**Supplementary Table 5c:** List of significantly differentially expressed genes between CSps and Kit K-MED.

**Supplementary Table 6:** Ingenuity molecular networks analysis gene list.
